# Hypoxia is correlated with the tumor immune microenvironment: Potential application of immunotherapy in bladder cancer

**DOI:** 10.1002/cam4.6617

**Published:** 2023-12-08

**Authors:** Haotian Chen, Yao Zhang, Xingyu Chen, Runshi Xu, Yuxing Zhu, Dong He, YaXin Cheng, Zhanwang Wang, Xiang Qing, Ke Cao

**Affiliations:** ^1^ Department of Oncology, Third Xiangya Hospital Central South University Changsha China; ^2^ Department of Pathology, Medical School Hunan University of Chinese Medicine Changsha China; ^3^ Department of Respiration The Second People's Hospital of Hunan Province of Hunan University of Chinese Medicine Changsha China; ^4^ Department of Otolaryngology—Head and Neck Surgery, Third Xiangya Hospital Central South University Changsha China

**Keywords:** bladder cancer, hypoxia, immune checkpoint inhibitor, TLR8, tumor microenvironment

## Abstract

**Objective:**

Hypoxia, which can considerably affect the tumor microenvironment, hinders the use of immunotherapy in bladder cancer (BLCA). Therefore, we aimed to identify reliable hypoxia‐related biomarkers to guide clinical immunotherapy in BLCA.

**Methods:**

Using data downloaded from TCGA‐BLCA cohort, we determined BLCA subtypes which divide 408 samples into different subtypes. Tumor immune infiltration levels of two clusters were quantified using ssGSEA, MCPcounter, EPIC, ESTIMATE, and TIMER algorithms. Next, we constructed a hypoxia score based on the expression of hypoxia‐related genes. The IMvigor210 cohort and SubMap analysis were used to predict immunotherapeutic responses in patients with different hypoxia scores. Hub genes were screened using cytoscape, immunohistochemistry (IHC), and multispectral immunofluorescence were used to detect the spatial distribution of immune markers.

**Results:**

Patients with BLCA were categorized into cluster1 (*n* = 227) and Cluster2 (*n* = 181). Immune infiltration and expression of immune markers were higher in Cluster1. Immune infiltration was also more obvious in the high‐hypoxia score group which related to a better predicted response to immunotherapy. IHC, and multispectral immunofluorescence confirmed the importance of TLR8 in immune infiltration and immune phenotype.

**Conclusions:**

BLCA subtype can evaluate the infiltration of immune cells in the tumor microenvironment of different patients. Hypoxia score in this study could effectively predict immunotherapeutic responses in patients with BLCA. TLR8 may be a potential target for clinical immunotherapy.

## 1 INTRODUCTION

Bladder cancer (BLCA) is the tenth most common cancer globally, with around 573,000 new cases and 213,000 deaths reported in 2020, characterized by a high risk of invasion and recurrence.^1^, ^2^ The emergence of immunotherapy has greatly improved the treatment mode of bladder cancer. Immune checkpoint inhibitors (ICIs) were studied in BLCA, revealing durable responses in some patients. ICIs such as programmed death ligand‐1 (PD‐L1) and programmed cell death protien‐1 (PD‐1) have received the most attention in the therapeutic area of BLCA.^3^, ^4^, ^5^ Despite the progress in the treatment, the number of people who see meaningful clinical benefit from ICI is limited.^6^, ^7^ Therefore, there is still a promising strategy for exploring better performing biomarkers allowing to improve prediction of response to ICIs.

Tumor microenvironment (TME) plays an important role in the development of resistance to ICIs.^8^ TME includes tumor cells, resident and recruited host cells, secretions from these cells and noncellular components and metabolites in the extracellular matrix.^9^ The tumor microenvironment is supplied with oxygen and nutrients by blood vessels and removes metabolic by‐products of cells. Blood vessels are one of the major pathways for immune cell trafficking and cancer cell dissemination. With rapid tumor growth, the original vasculature of the tumor tissue is not adequately perfused and, as a result, decreased oxygen levels establish a hypoxic and malnourished environment and hypoxia becomes a hallmark manifestation of solid malignancies.^10^, ^11^

Hypoxia is one of the central players in shaping the tumor immune microenvironment (TIME) in which metabolic by‐products and immunosuppressive modulators accumulate.^9^, ^12^ Multiple metabolic interactions between tumor and stromal cells and hypoxia‐induced angiogenesis contribute to the prevalence of immunosuppression in the hypoxic tumor microenvironment.^13^ Hypoxia significantly induces the generation of an immunosuppressive environment and ultimately impairs immune cell adaptation and function.^14^ For example, hypoxia causes apoptosis of CD8^+^ T lymphocytes and reduces their recruitment to the tumor.^15^ In conclusion, hypoxia inhibits immune activation in the antitumor process on the one hand and promotes tumor immune escape on the other. In a hypoxic environment, cells sensitive to immunotherapy may turn into drug‐resistant cells, making tumor cells resistant to ICIs, which is an important cause of immunotherapy failure.^16^ Therefore, the exploration of hypoxia‐related biomarkers may provide new ideas for immunotherapy in bladder cancer.

Bioinformatics analysis provides a useful tool to evaluate the responsiveness to immunotherapy from transcriptional data, genomic data, and clinical information. Song et al.^17^ used unsupervised clustering to classify BLCA patients into four subtypes and determined prediction of response to ICIs. Chen et al.^18^ identified genes significantly associated with CD8^+^ T effector and immune checkpoint signatures that effectively predict prognosis and responsiveness to immunotherapy in patients with bladder cancer. In the present study, we classified the BLCA patients into two clusters and constructed the hypoxia score based on hypoxia‐related genes for predicting ICI treatment responsiveness, and hub gene *TLR8* can be a suitable target for clinical immunotherapy in BLCA.

## 2 MATERIALS AND METHODS

### 2.1 Data acquisition and processing

Complete clinical information along with RNA sequencing, somatic mutation, and copy number variation (CNV) data of 408 BLCA cases were downloaded from The Cancer Genome Atlas (TCGA; TCGA‐BLCA cohort) using the “TCGAbiolinks” R package.^19^ We excluded genes with zero expression and samples without clinical survival information. mRNA expression was quantified as FPKM. Hypoxia‐related genes were obtained from the GeneCards database (https://www.genecards.org/; version 5.0). We also accessed the cohort from IMvigor210, a single‐arm, multicenter phase II study evaluating the clinical activity, safety, and efficacy of the PD‐L1 inhibitor atezolizumab in urothelial carcinoma. Full expression data and detailed clinical information for this cohort is available from the “IMvigor210CoreBiologies” R package under the Creative Commons 3.0 license.^20^ Detailed information of TCGA‐BLCA dataset and IMvigor210 cohort are summarized in Tables S1 and S2.

### 2.2 Weighted gene co‐expression network construction

A scale‐free gene co‐expression network of BLCA was established by setting the soft threshold *β* = 4 and *R* ^2^ = 0.91 using the R package “WGCNA”.^21^ The power function was used to transform the gene expression matrix into an adjacency matrix and then the adjacency matrix into a topological overlap matrix to measure the connectivity of the gene network. The “cutreeDynamic” function was used to prune the gene hierarchical clustering dendrogram to generate co‐expression modules. Next, we classified genes with similar expression profiles into different modules, wherein the module eigengenes (MEs) of the modules represented the gene expression profiles in their corresponding modules. Finally, we used Pearson's correlation analysis to identify modules that were highly related to immune cell infiltration.

### 2.3 Identification and validation of hub genes

Node genes that are closely related to other nodes in a module are defined as “hub genes” and are considered to have important biological functions. Cytoscape plugins, “MCODE” and “cytoHubba”^22^, ^23^, ^24^ were used to determine the interactive relationship of hub genes in the network, and hub genes were screened as per the degree algorithm. GSCALite,^25^ a web‐based analytics platform, was used for the pan‐cancer analysis of hub genes. Immune analysis of the hub genes was performed using SangerBox, a free online platform for data analysis.

### 2.4 Consensus clustering of the BLCA subtypes

A consensus clustering algorithm was applied to determine the number of clusters in TCGA‐BLCA cohort using the R package “ConsensusClusterPlus”.^26^ To ensure the stability of the classification, the algorithm is repeated 50 times (resampling rate = 80%). “Limma” R package^27^ was then used to identify differentially expressed genes (DEGs) among the different subtypes of BLCA for subsequent analyses. The cutoff criteria for determining DEGs were set to corrected *p* < 0.05 and |logFC| > 1. There are several molecular subtype systems: Baylor, University of North Carolina at Chapel Hill (UNC), MD Anderson Cancer Center (MDA), TCGA, Cartes d'Identite des Tumeurs (CIT)‐Curie, Lund, and consensus classification.^28^, ^29^, ^30^, ^31^, ^32^, ^33^, ^34^ Similarities exist between different molecular types. The BLCA molecular subtypes in this study were compared with those published bladder cancer molecular subtypes.

### 2.5 Functional and pathway enrichment analyses

For the functional enrichment analysis, genes in specific modules and DEGs were mapped to the terms in Gene Ontology (GO) and Kyoto Encyclopedia of Genes and Genomes (KEGG) databases. GO analysis The GO analysis includes terms rich in biological process (BP), cellular component (CC), and molecular function (MF). The cut‐off values for GO and KEGG term selection were *p* < 0.01 and FDR < 0.05. To identify the differentially activated signaling pathways between the different BLCA subtypes, gene set enrichment analysis (GSEA) was used. The gene set was obtained from the hallmark gene sets in MSigDB. The above functional annotation analysis was finished using the “clusterProfiler” R package.^35^

### 2.6 Comparison of mutational differences and CNVs

The determination the mutational profile of different subtypes was finished by the R package “maftools”.^36^ Mutation rates can be estimated for each gene and important mutated genes in different clusters can be identified (*p* < 0.05). GISTIC 2.0^37^ was used to assess the number of copies that were significantly amplified or deleted. The loss or increase in burden was calculated as the total number of genes with copy number changes at the focal and arm levels. The effect of CNVs of the hub genes on immune cell infiltration levels was evaluated using the Tumor IMmune Estimation Resource (TIMER)^38^ which includes six immune cell types such as B cells and T cells. Data from GISTIC 2.0 were used in TIMER.

### 2.7 Comparison of enriched oncogenic pathways

We collected 12 BLCA signatures, specific to different molecular subtypes and immunotherapy‐associated gene signatures, from previous publication.^34^, ^39^ We then used ssGSEA to generate enrichment scores for each pathway in each sample. Subsequently, we compared the ssGSEA scores for each pathway using the “GSVA” R package.^40^ In addition, we collected 10 oncogenic pathways associated with BLCA,^41^ and using ssGSEA, we generated an enrichment score. We subtracted the inhibition score from the activation score to obtain the final enrichment score. This was also implemented using the GSVA package.

### 2.8 Landscape of the BLCA immune microenvironment

Download the list of genes in the seven steps of anticancer immune response from TIP (//biocc.hrbmu.edu.cn/TIP/index.jsp). Scores of the seven steps were obtained using single‐sample GSEA (ssGSEA) and the scores of different subtypes were compared.^42^ To explore the relationship between the different clusters and the immune microenvironment of BLCA, we quantified the infiltration levels of different immune cells. Due to the use of different algorithms and marker gene sets can lead to calculation errors, we used five independent algorithms, ssGSEA, MCPcounter, EPIC, ESTIMATE, and TIMER,^38^, ^42^, ^43^, ^44^, ^45^ to calculate the infiltration levels of the immune cells. ssGSEA applies the gene characteristics expressed by the immune cell population to a single tumor sample, and the standardized gene expression data are then used to infer the relative proportion of the TME immune‐infiltrating cells. The gene sets for 28 immune cell types are from the study of Charoentong et al.^46^ The MCPcounter is used to steadily quantify the absolute abundance of eight types of immune cells and two types of stromal cell populations from transcriptome data. EPIC was used to estimate the proportion of cancer and immune cell types. ESTIMATE calculates the stromal and immune scores to predict the level of tumor stroma and immune cells; the “ESTIMATE score,” which is the sum of the immune and stromal scores, is used for inferring tumor purity in tumor tissues. A higher score of immune or stromal indicates more immune or stromal components in the TME, respectively. Additionally, the abundance of six types of tumor‐infiltrating immune cells is systematically recorded in TIMER. Furthermore, to evaluate the immune microenvironment infiltration in different patients more intuitively, we obtained H&E‐stained images of BLCA samples of TCGA‐BLCA from the study by Robertson et al.^31^

### 2.9 Dimensionality reduction and generation of hypoxia score

Because of the complexity and heterogeneity in the TME of patients, we conducted PCA to construct a hypoxia score. PCA has the advantage of focusing scores on sets with the largest blocks of related (or anti‐related) genes in a given set, while reducing the weight of contributions from genes that are not related to other set members. This method uses dimensionality reduction to convert multiple evaluation indicators into one evaluation indicator, that is, a linear transformation. We then defined the hypoxia score using a method similar to that of gene expression grade index (GGI)^47^, ^48^:

$$\text{hypoxia score}=\sum \left(\mathrm{PC}1i+\mathrm{PC}2i\right),$$

where *i* is the expression of the hypoxia‐related genes. Both principal components 1 and 2 (PC1 and PC2) were selected as hypoxia scores.

### 2.10 Prediction of the immunotherapeutic response

Based on the study by Mariathasan et al.,^20^ we evaluated the relationship between different hypoxia scores of BLCA in immune‐related pathways, and evaluated their effectiveness in response to ICI, including PD‐L1 expression level on immune cells and PD‐L1 expression level on tumor cells, response to immune checkpoint blockade (ICB), tumor mutation burden (TMB), and mutation status for genes of interest. Next, we analyzed the differences in the expression of key biological pathways in bladder cancer between the different hypoxia score groups, using a heatmap to visualize the results of these analyses. We then used SubMap analysis to determine the efficacy of using anti‐PD‐1 and anti‐CTLA4 in patients with different hypoxia scores to improve the accuracy of clinical outcome predictors.^49^

### 2.11 Cell culture and cell proliferation assay

Human BLCA cells (T24) were obtained from American Type Culture Collection (ATCC), culturing in 10% fetal bovine serum (VS500T, Ausbian) supplemented RPMI 1640 medium (Corning, NY, USA) and cultured at 37°C in a 5% CO_2_ incubator (Sanyo, Osaka, Japan). The cell suspension (approximately 3–5 × 10^3^) was plated into 96‐well plates 1 day before lentivirus infection. HCS automates image acquisition and analysis in microscopy. The HCS method was used to perform the cell proliferation assay. Three RNA interference targets were designed for a gene, and three plasmids carrying different targets were packaged by mixed lentivirus. The sequences for each short hairpin RNA (shRNA) are listed in Table S3. At the same time, the ShCtrl group (non‐targeting shRNA) were generated. On the day of infection, an appropriate quantity of lentiviral particles was added according to the multiplicity of infection of the cells to infect the target cells. After 2–3 days of infection, green fluorescence protein expression was observed under a fluorescence microscope, and cells were collected when the cell density reached 80%. Celigo imaging cytometer (Nexcelom Bioscience LLC, Lawrence, MA, USA)^50^ was used to capture live cells, daily, for 5 days. The cells with green fluorescence were identified and their corresponding images were saved and analyzed to calculate the number of cells in different groups. After continuous observation for 5 days, cell growth curves were plotted to determine the cell proliferation.

### 2.12 Immunohistochemistry, and multispectral immunofluorescence of BLCA samples

BLCA tissues were obtained from 16 patients who underwent surgery at the Department of Urology of the Third Xiangya Hospital (Changsha, China). BLCA tumor tissue was formalin‐fixed paraffin‐embedded. The study was approved by the Ethics Committee of the Third Xiangya Hospital, Central South University, and informed written consent was obtained from all the patients. The tissue samples were used for immunohistochemical analysis. CD8‐specific antibodies (1:150, AF20211, afantibody), anti‐PD‐L1 antibody (1:150, AF20084, afantibody), and anti‐TLR8 antibody (1:100, DF6426, Affinity) were used in combination with a secondary antibody which is horseradish peroxidase [HRP]‐conjugated goat anti‐rabbit IgG. Freshly prepared 3,3′‐diaminobenzidine tetrahydrochloride reagent (G1211, Servicebio) was used for staining. For staining of CD8, PD‐L1, and TLR8, we calculated the percentage of cells which have strong staining intensity of the membrane (brown staining). Due to the spatial distribution of the CD8^+^ T cells, tumors were categorized into three phenotypes: “immune inflamed,” “immune excluded,” and “immune desert.” The cells were considered immune inflamed when they were located in the tumor parenchyma and stroma surrounding the tumor; immune excluded when they were located in the stroma, but not in the parenchyma; and immune desert when they were absent from both the tumor parenchyma and stroma. As described by Chen et al.,^51^ we performed multispectral immunofluorescence staining. Staining of slides to allow simultaneous visualization of seven markers Abs anti‐CK19 (1:400, Cat# ab52625, Abcam), anti‐TLR8 (1:50, DF6426, Affinity), anti‐PD‐L1 (Cat# ab213524, Abcam), anti‐CD8 (1:200, Cat# ab101500, Abcam), anti‐PD‐1 (1:100, UMAB199, Origene), anti‐foxp3 (1:200, Cat# ab215206, Abcam), anti‐CD56 (1:250, Cat#ab133345, Abcam), incubating at 47°C overnight. The secondary antibody was Opal polymer HRP, and Opal 7 Color Kit (PerkinElmer, Hopkinton, MA, USA) was used. seven staining cycles were performed: anti‐CK19/Opal‐520, anti‐TLR8/Opal‐650, anti‐PD‐L1/Opal‐700, anti‐CD8/Opal‐570, anti‐PD1/Opal‐620, anti‐foxp3/Opal‐540, and anti‐CD56/Opal‐440. Finally, we used 4′‐6′‐diamidino‐2‐phenylindole (DAPI) to highlight all nuclei. Scanning of stained sections, acquisition of multispectral images and spatial analysis of multispectral imaging data were conducted by the TissueFAXS platform and the StrataQuest software (TissueGnostics, Vienna, Austria).

### 2.13 Statistical analysis

Unpaired *t*‐test was applied to compare the normally distributed variables between two groups, and the Wilcoxon rank‐sum test was used to evaluate the significance of variables that were not normally distributed. Analysis of variance (ANOVA) and Kruskal–Wallis tests were used to estimate the differences in three or more groups. The chi‐squared (*χ* ^2^) test was used to analyze categorical variables. Pearson's correlation analysis and Spearman's correlation analysis were used for the calculation the correlation between two variables. The Kaplan–Meier method was used to analyze the prognosis and the logrank test was used to determine significant differences. A two‐way ANOVA was used to evaluate the significance of the cell proliferation assay. All the above statistical analyses were performed using R (https://www.r‐project.org/) and GraphPad Prism 8.0 (GraphPad Software, San Diego, CA). Two‐sided *p* < 0.05 was considered significant.

## 3 RESULTS

### 3.1 WGCNA construction and key module identification

**FIGURE 1:**
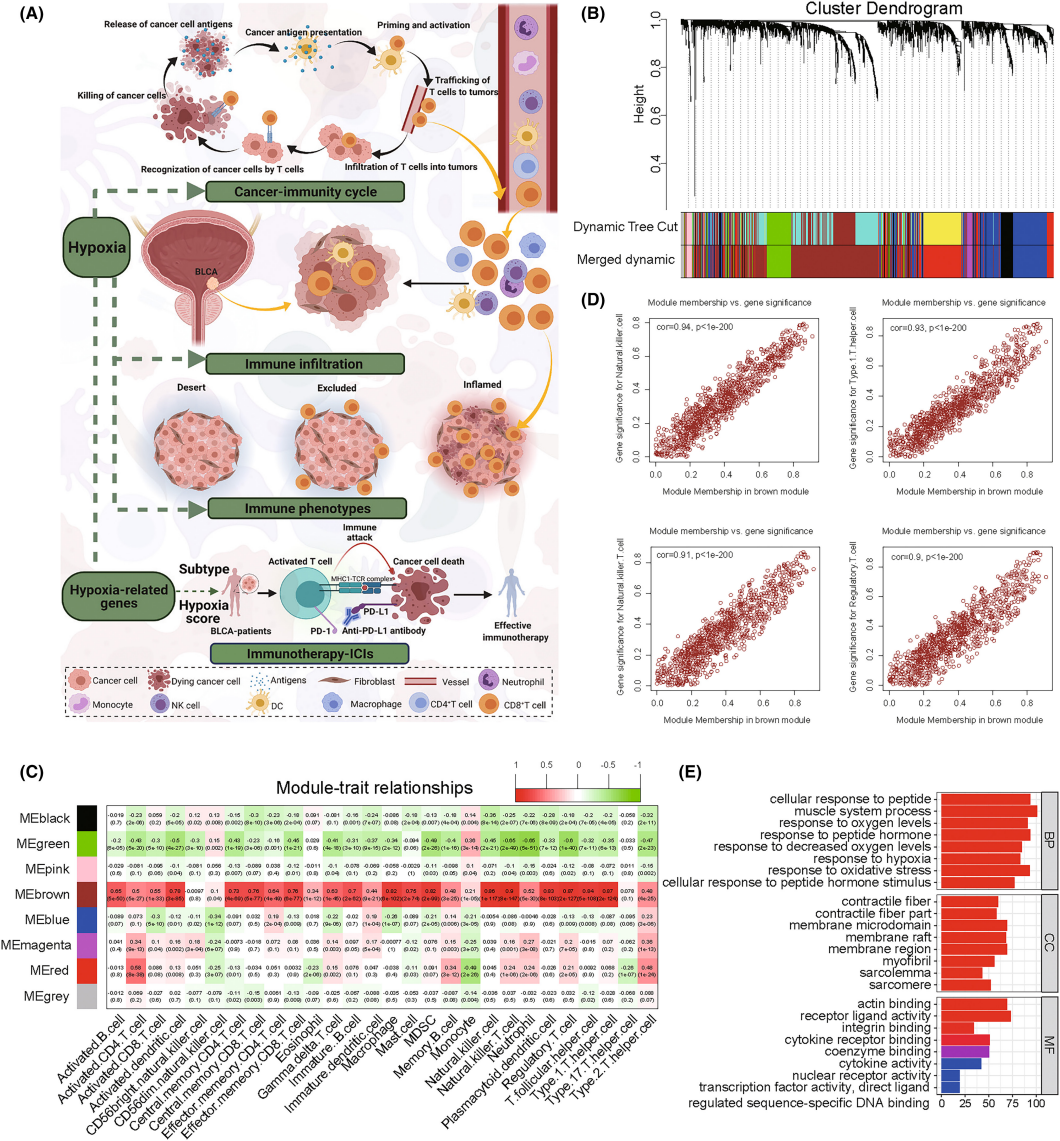
Weighted gene co‐expression network for bladder cancer (BLCA). (A) Landscape of the impact of hypoxia on immune infiltration and immunotherapy in BLCA. The picture was created with BioRender.com. (B) Cluster dendrogram of hypoxia‐related genes in the co‐expression network. The branches of the cluster dendrogram correspond to different modules. Each portion of the dendrogram leaf corresponds to a gene. (C) Heatmap shows the correlation between the modules and 28 immune cells, with the brown module (BM) showing the most positive correlation with the immune cells. Each row corresponds to a module eigengene (ME), and each column corresponds to the immune cells. Each cell contains the corresponding correlation value and p‐value, with color depth indicating decrease in the correlation value. Red indicates positive correlation, and green indicates negative correlation. (D) Scatter plot of the correlation between module membership (MM) in the BM and gene significance (GS) for NK cells, Th1 cell, NKT cells, and regulatory T cells. (E) Gene ontology (GO) enrichment analysis of hypoxia‐related genes in the BM. The x‐axis represents the number of genes in each GO item, and the y‐axis represents the biological process. Color grading red to blue indicates decrease in the p‐value.

The study workflow is shown in Figure S1. Figure 1A summarizes the possible mechanism by which hypoxia affects immune infiltration process and the immunotherapy responsiveness in BLCA. 517 hypoxia‐related genes from the 408 BLCA samples were selected. The co‐expression network was constructed and confirmed to be a scale‐free network (Figure S2A,B). The first set of modules was obtained by the use of the dynamictreecut algorithm; all related modules were merged to generate several modules, which were represented by different colors. The number of genes in the setting module was >30 (Figure 1B). In each module, gene co‐expression was summarized by the eigengenes (the first component expression of genes belonging to a module). Among the eight modules selected, the MEs of the brown module (BM) showed the strongest correlation with 28 types of immune cells (Table S11). BM is highly associated with immune cells. For instance, the correlation coefficient between NKT cells and BM was 0.9 (*p* = 8e−147; Figure 1C), so we considered it as the key module. To explore the association between hypoxia and immune cells, we plotted a scatter plot of gene significance (GS) versus module membership (MM) in BM, where there was a highly significant correlation between GS in immune cells and MM in BM. The results showed a significant positive correlation of hypoxia‐related genes with NK cells, Th1 cells, NKT cells, and regulatory T cells, etc. (all *p* ≤ 3.1e−98; Figure 1D; Figure S2C). The hypoxia‐related genes were enriched in “cellular response to peptide,” “muscle system process,” “response to peptide hormone,” etc. in BP; “membrane microdomain,” “membrane raft,” “membrane region,” etc. in CC; and “actin binding,” “receptor ligand activity,” “cytokine receptor binding,” etc. in MF (Figure 1E; Table S4). KEGG analysis reveals that depleted oxygen‐related genes in the brown module are associated with cancer development and immune response pathways such as “PI3K‐Akt signaling pathway,” “human cytomegalovirus infection,” and “proteoglycans in cancer” (Figure S2D; Table S5).

### 3.2 Classification of BLCA subtypes by consensus clustering

**FIGURE 2:**
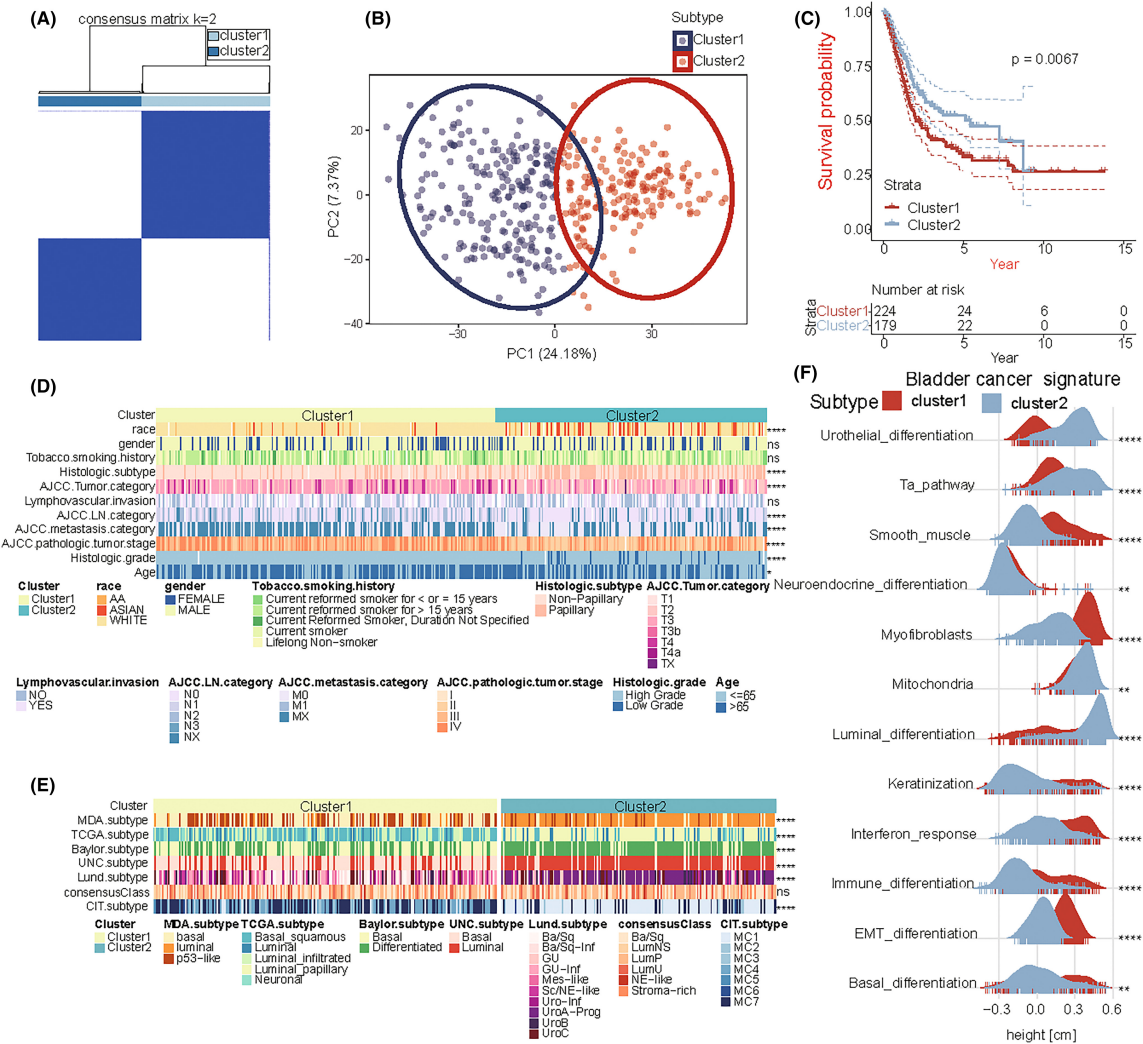
Clustering of BLCA subtypes based on the expression of hypoxia‐related genes in the brown module (BM). (A) The cluster stability was evaluated using the “ConsensusClusterPlus” R package, and the consensus cluster matrix for *k* = 2 revealed two major clusters. Total 408 BLCA cases in the Cancer Genome Atlas (TCGA) were categorized into two subtypes: Cluster1 (*n* = 227) and Cluster2 (*n* = 181). (B) Principal component analysis (PCA) confirmed that the cases could be classified into Cluster1 and Cluster2. (C) Kaplan–Meier curves of overall survival of patients with BLCA in the two clusters (log‐rank test, *p* = 0.0067). (D) Heatmap of the clinicopathologic features of the two clusters. Significance is denoted by asterisks (chi‐square test; ****p < 0.0001, *p < 0.05, ns, not significant). (E) Heatmap of the relationship between the subtypes generated in this study and the existing BLCA subtypes. Significance is denoted by asterisks (chi‐squared test; ****p < 0.0001, ns, not significant). (F) Score distributions of 12 BLCA signatures between the two clusters. Significance is denoted by asterisks (Wilcoxon rank‐sum test; ****p < 0.0001, **p < 0.01).

Unsupervised clustering methods were used to classify patients with BLCA for further analysis. According to the similarity shown by the expression of hypoxia‐related genes, *k* = 2 was considered for an optimal clustering stability (Figure 2A; Figure S3A,B). Total 408 patients with BLCA were classified into two subtypes, Cluster1 (*n* = 227) and Cluster2 (*n* = 181). PCA confirmed that these genes can effectively divide BLCA patients to two clusters (Figure 2B). Survival analysis was performed on samples with prognostic information, and the results showed that the overall survival of Cluster1 was significantly shorter than that of Cluster2 (log‐rank test, *p* = 0.0067; Figure 2C). In the clinical features, including race, histologic subtype, and tumor‐node‐metastasis stage, etc. between the two clusters, there were significant differences (chi‐squared test, *p* < 0.05); however, no significant differences were observed in gender, history of smoking, and lymphovascular invasion between the two clusters (Figure 2D). Similarities exist between different molecular subtypes. On comparing this classification with existing, published classifications, we observed that Cluster1 and Cluster2 were, respectively, similar to the basal and luminal subtypes in the MDA classification; basal‐squamous and luminal‐papillary subtypes in TCGA classification; basal and differentiated subtypes in the Baylor classification; basal and luminal subtypes in the UNC classification; Ba/Sq and UroA‐Prog subtypes in the Lund classification; and MC7 and MC1 subtypes in the CIT classification (chi‐squared test, *p* < 2.2e−16). However, the extent of difference between the two clusters was small in the consensus classification (Figure 2E; Figure S3C). The score of 12 BLCA signatures of the two clusters was calculated using ssGSEA. Subsequently, we observed higher enrichment scores in Cluster1 for smooth muscle, myofibroblasts, interferon response, and immune differentiation, and in Cluster2 for urothelial differentiation, TA pathway, and luminal differentiation (Figure 2F).

### 3.3 Functional annotation of the BLCA subtypes

**FIGURE 3:**
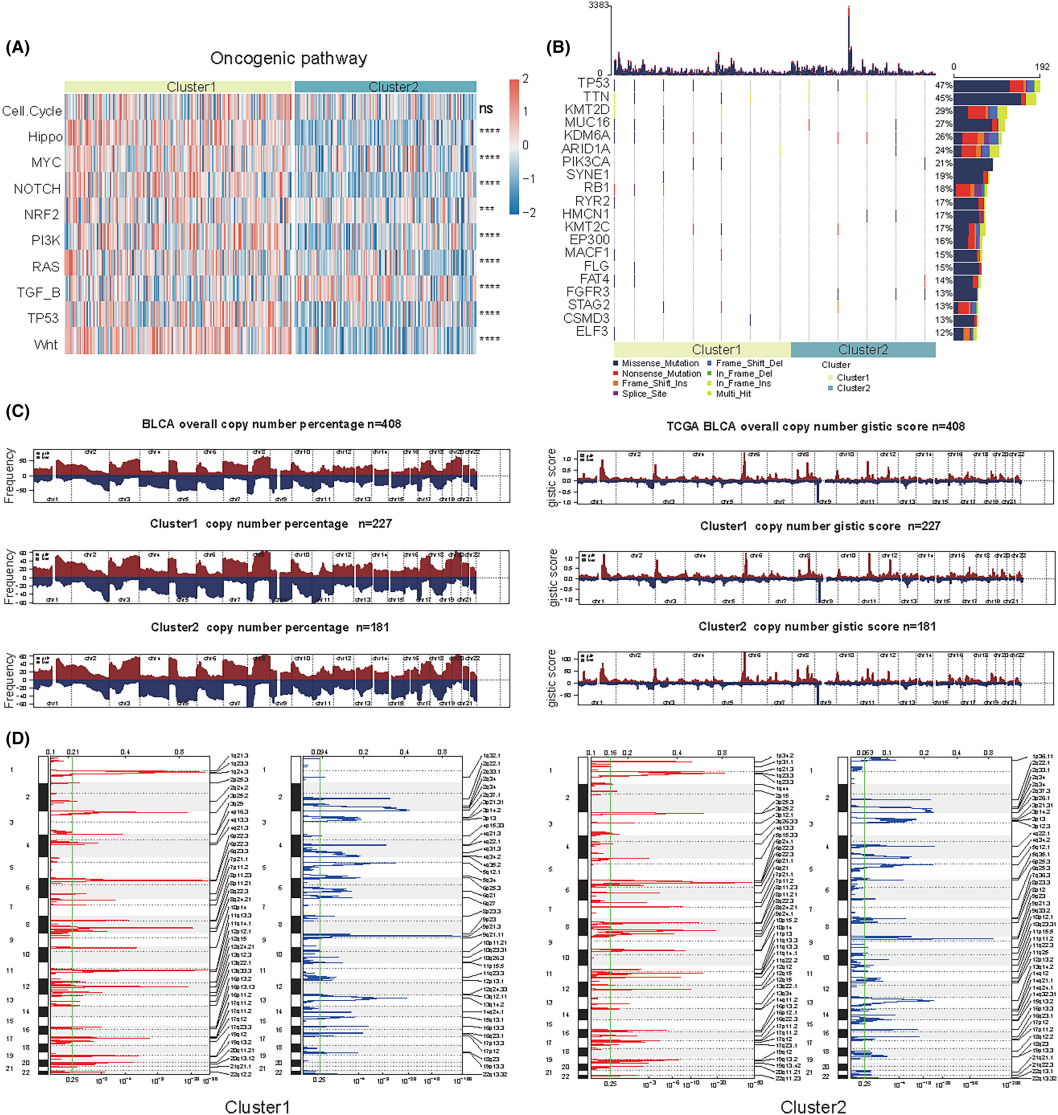
Functional annotation and genomic alterations. (A) Heatmap showing the differences in the enrichment scores of 10 oncogenic pathways in the two clusters. Significance is denoted by asterisks (Wilcoxon rank‐sum test; ****p < 0.0001, ***p < 0.001, ns, not significant). (B) The waterfall plot shows mutations in the top 20 genes with the highest mutation frequency. The number on the right is the mutation frequency of each gene, and the right side of the number depicts the mutation type and mutation proportion. Different colors represent different mutation types. (C) The plot on the left illustrates the frequency of gain (dark red) and loss (dark blue) of each gene. The plot on the right illustrates the GISTIC score of the gain (dark red) and loss (dark blue) of each gene. Gene segments are placed according to their location on chromosomes 1–22. (D) False‐discovery rates (*q*‐values) and GISTIC 2.0 scores for alterations (*x*‐axis) are plotted against the genome positions (*y*‐axis); the dotted lines indicate the centromeres. The amplifications (red) and deletions (blue) of the two clusters are also shown. The *q*‐value level of each locus has been plotted.

We calculated the enrichment scores of the 10 most common oncogenic pathways of BLCA between the two clusters and observed that most pathways had higher enrichment scores in Cluster1 than in Cluster2 (*p* < 0.001; Figure 3A). Blocking these oncogenic pathways may benefit Cluster1 patients. To further estimate the significance of DEGs in the regulation of the BLCA immune microenvironment, we analyzed the biological pathways. GO analysis showed that the significantly enriched BP terms were “extracellular matrix organization,” “extracellular structure organization,” and “regulation of lymphocyte activation,” etc.; CC terms were “collagen‐containing extracellular matrix,” “external side of plasma membrane,” and “endoplasmic reticulum lumen,” etc.; and MF terms were “extracellular matrix structural constituent,” “glycosaminoglycan binding,” and “sulfur compound binding,” etc. (Figure S4A; Table S6). KEGG analysis revealed that these DEGs were mainly enriched in “cytokine‐cytokine receptor interactions” (Figure S4B; Table S7). GSEA analysis showed that the DEGs were closely correlated with “complement,” “EMT,” “JAK‐STAT3 signaling via IL6,” “inflammatory response,” and “interferon‐gamma response” (Figure S4C; Table S8).

### 3.4 Genomic alterations of the BLCA subtypes

Next, we investigated the somatic mutations of DEGs between the two clusters and identified mutations in top 20 genes, such as TP53, TIN, KMT2D, MUC16, KDM6A, ARID1A, PI3KCA, SYNE1, and RB1, which were considered to be important in immunotherapy and tumor development of bladder cancer (Figure 3B).

Additionally, we analyzed the CNVs data of the two subtypes, showed the difference in the distribution of copy number changes between the two subtypes, identified the frequency of gene amplification and depletion, and calculated the gistic score, that is, the G‐score. The higher the G‐score, the more likely copy number variation event will occur in that region. The parameter threshold was defined as *p* < 0.05 for amplified or depleted fragment lengths >0.1(Figure 3C). GISTIC revealed that Cluster1 had 41 copy number amplified regions and 42 copy number depleted regions, while in Cluster2 there were 50 copy number amplified regions and 47 copy number depleted regions. For example, the significantly amplified regions in Cluster1 are located on chromosomes 6p, 6q, 11q, etc., and the significantly depleted regions are 9p21.3, etc. Significantly amplified regions in Cluster2 are located on chromosome 6p, etc., and significantly depleted regions are located on chromosome 9p, etc. (Figure 3D). The total copy number variation profiles of the two subtypes were displayed with a heatmap, and we can see that there are differences in the copy number variation between the two subtypes. For example, the amplification events in the chromosome 8 region and the depletion events in the chromosome 10 region in Cluster1 are more obvious than those in Cluster2 (Figure S4D).

### 3.5 TME cell infiltration characteristics in the BLCA subtypes

**FIGURE 4:**
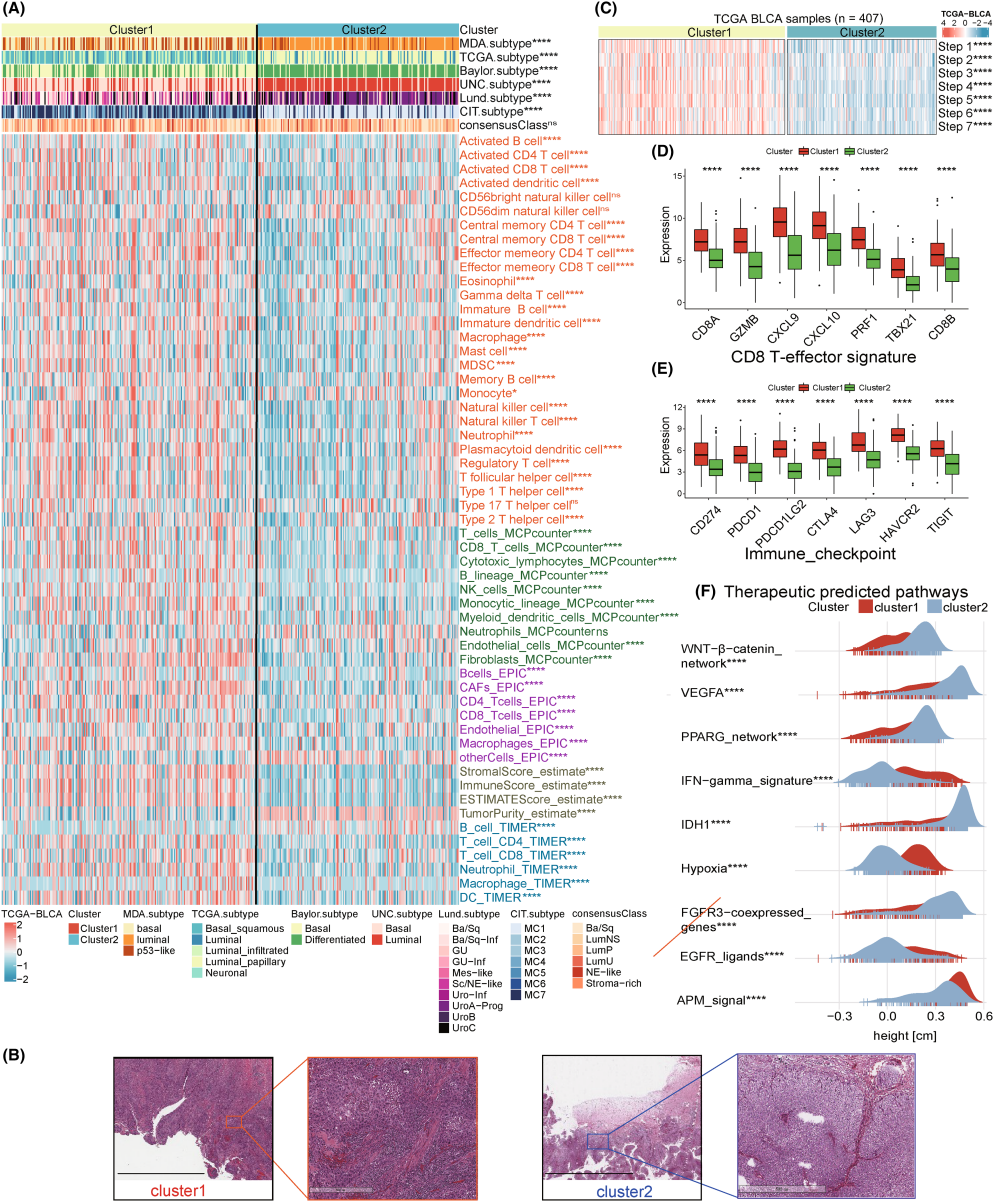
Tumor microenvironment (TME) cell infiltration characteristics in the two clusters. (A) The heatmap shows the differences in immune infiltration between the two clusters (Wilcoxon rank‐sum test; ****p < 0.0001, *p < 0.05, ns, not significant). (B) Differences in immune cell infiltration in BLCA sections downloaded from TCGA. In the left images of two clusters, the scale bar corresponds to 10 μm and in the right, it corresponds to 500 μm. (C) The ssGSEA algorithm was used to obtain the scores of seven steps of the cancer‐immunity cycle. Significance is denoted by asterisks (Wilcoxon rank‐sum test; ****p < 0.0001). (D, E) The box plot shows the expression of CD8 T‐effector signature and immune checkpoints in the two clusters. The ends of the box represent the upper and lower quartiles, the line in the box represents the median value, and the black dots represent the outliers. Significance is denoted by asterisks (Wilcoxon rank‐sum test; ****p < 0.0001). (F) The mountain plot shows the differences in the enrichment scores of immunotherapeutic predicted pathways between the two subtypes. Significance is denoted by asterisks (Wilcoxon rank‐sum test; ****p < 0.0001).

Using ssGSEA, we compared the differences in the abundance of 28 immune cells between the two clusters and found that the infiltration density of the immune cells in Cluster1 are significantly higher than that in Cluster2. To reveal the role of the subtypes based on hypoxia‐related genes in the TME, we used the ESTIMATE algorithm to estimate the ratio of the immune and stromal cells and quantified the overall infiltration. This algorithm evaluates three scores: stromal score, immune score, and ESTIMATE score. ESTIMATE scores were higher in Cluster1 than in Cluster2, suggesting differences in the TME‐infiltrating cells between the two subtypes, and that hypoxia‐related genes may play a role in immune regulation. In addition, the MCPcounter, EPIC, and TIMER algorithms verified these observed differences. The comparison of the two subtypes with the existing molecular subtypes and immune infiltration differences are presented in a heatmap (Wilcoxon rank‐sum test, Figure 4A). We further verified this via the infiltration observed in the pathological sections of BLCA samples in TCGA cohort^31^ (Figure 4B). Next, we used the ssGSEA algorithm to obtain the scores and differences in the scores for each cancer‐immunity cycle step between the two clusters. For the anticancer immune response to eliminate cancer cells, a series of stepwise events must be initiated, allowed to proceed, and expand iteratively. These steps constitute the cancer‐immunity cycle, and include the following seven steps: the release of cancer cell antigens, cancer antigen presentation, priming and activation, trafficking of T cells to tumors, infiltration of T cells into tumors, recognition of cancer cells by T cells, and killing of the cancer cells.^52^ Cluster1 had higher scores than that in Cluster2 in the seven steps (Wilcoxon rank‐sum test, *p* < 0.0001; Figure 4C). To explore the regulation and effect of the different subtypes on immunity, we analyzed the expression of CD8 T‐effector signature and related immune checkpoints.^20^ The results showed that the expression of these genes in Cluster1 was also significantly higher than that in Cluster2 (*p* < 0.0001; Figure 4D,E). Furthermore, the enrichment scores of immunotherapy pathways, such as IFN‐γ signature, hypoxia, and APM signaling, were higher in Cluster1. Whereas, the scores such as Wnt‐β‐catenin signaling, VEGFA, PPARG network were lower in Cluster1. Blocking these oncogenic pathways may benefit Cluster2 patients (*p* < 0.0001; Figure 4F).

### 3.6 Construction of the hypoxia score

**FIGURE 5:**
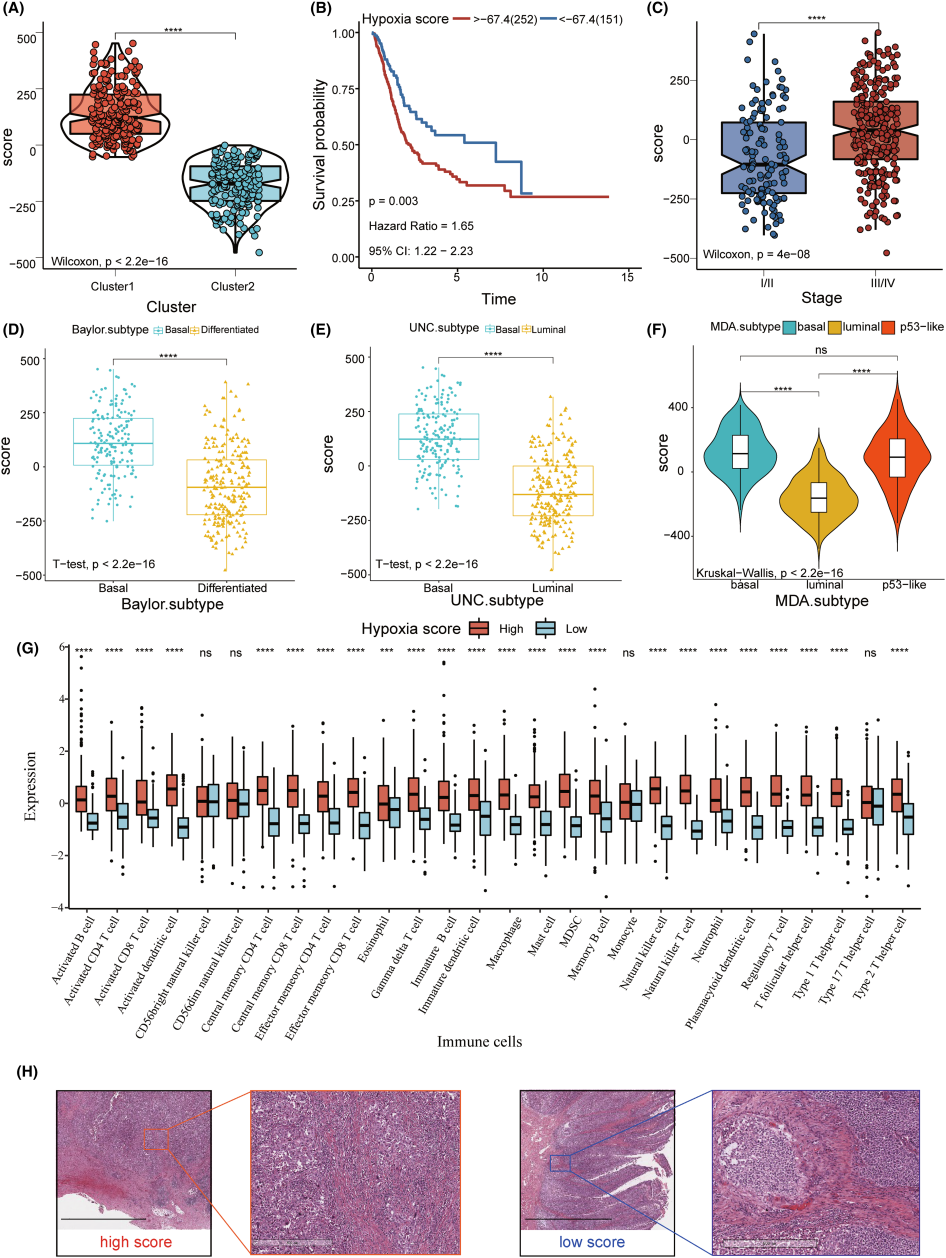
Construction of the hypoxia score. (A) Hypoxia score of Cluster1 was higher than that of Cluster2 (Wilcoxon rank‐sum test; *p* < 2.2e−16). (B) Kaplan–Meier curve shows that the overall survival (OS) of patients with high scores is significantly shorter than that of patients with low scores (log‐rank test; *p* = 0.003). (C) Scores of clinical stages III and IV were higher than those of I and II (Wilcoxon rank‐sum test; *p* = 4e−08). (D, E) Differences in the hypoxia scores in the Baylor and UNC subtypes (*t* test, *p* < 2.2e−16). (F) Differences in the hypoxia score in the MDA subtype (Kruskal–Wallis; *p* < 2.2e−16). (G) Infiltration levels of 28 immune cell types in high and low score groups. Horizontal and vertical axes represent immune cells and relative percentages, respectively. The ends of the box represent the upper and lower quartiles, the line in the box represents the median value, and the black dots represent the outliers. Significance is denoted by asterisks (Wilcoxon rank‐sum test; ****p < 0.0001, ns, not significant). (H) Images of pathological sections of TCGA‐BLCA samples show the differences in immune cell infiltration between the high and low score groups. The scale bars correspond to 10 μm in the left images of two hypoxia score group, and to 500 μm in the right.

To evaluate the immune microenvironment of each patient and determine reliable and effective biomarkers to predict immunotherapeutic response, we constructed and evaluated a hypoxia score based on the expression levels of the hypoxia‐related genes in the BM. Cluster1 had a significantly higher hypoxia score (Wilcoxon rank‐sum test, *p* < 2.2e−16; Figure 5A). Accordingly, the patients were categorized into high‐score and low‐score groups. Survival analysis revealed that the prognosis of patients in the high‐score group was worse than that in the low‐score group (log‐rank test, hazard ratio [HR] = 1.65, 95% confidence interval [CI] = 1.22–2.23, *p* = 0.003; Figure 5B). The scores of clinical stages III and IV were higher than those of I and II (Wilcoxon rank‐sum test, *p* = 4e−08; Figure 5C; Table S9). Next, we analyzed the differences in the hypoxia scores among the existing BLCA molecular subtypes and found that the score of basal was higher than the score of differentiated in the Baylor classification, the score of basal was higher than the luminal score in the UNC classification, and the score of basal was the highest, followed by p53‐like, and luminal in the MDA classification (*p* < 2.2e−16; Figure 5D–F). Next, we used ssGSEA to compare the differences in the abundance of the 28 immune cells in the high‐score and low‐score groups and found that immune cell infiltration in the high‐score group was stronger than that in the low‐score group (Figure 5G). This is consistent with a difference in immune infiltration in both clusters. The pathological sections from TCGA‐BLCA cohort also confirmed this immune cell infiltration^31^ (Figure 5H). Furthermore, Pearson's correlation analysis, which was used to evaluate the correlation between the hypoxia score and immune checkpoints, showed a high positive correlation (Figure S5).

### 3.7 Validation of the benefits of immunotherapy

**FIGURE 6:**
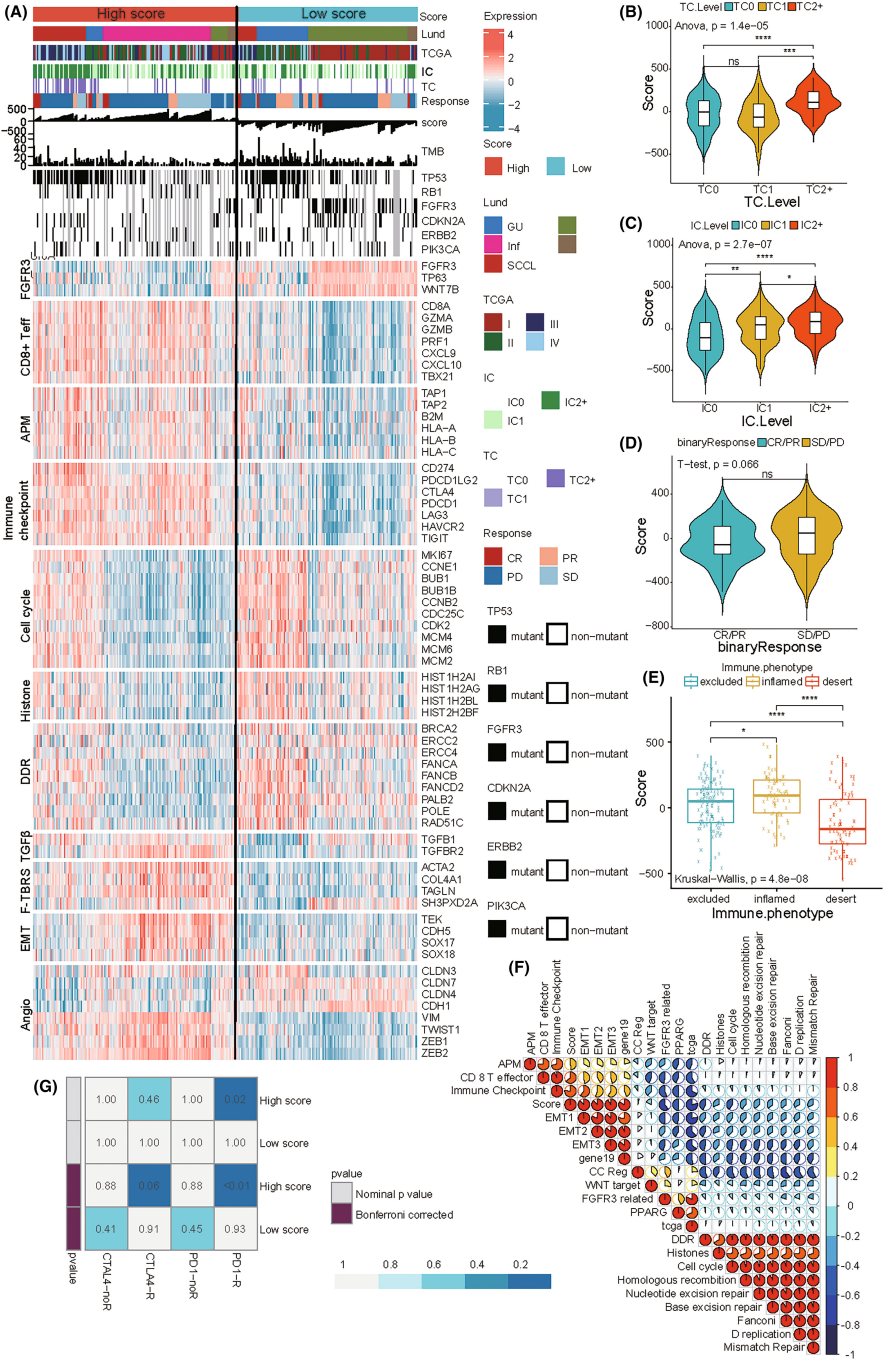
IMvigor210 cohort for immunotherapy prediction. (A) The heatmap shows the effects of hypoxia score on PD‐L1 treatment in the IMvigor210 cohort. IC: PD‐L1 expression on tumor cells and TC: PD‐L1 expression on immune cells (TC2+ ≥ 50%, TC2 ≥ 5% and <50%, TC1 ≥ 1% and <5%, and TC0 < 1%; IC3 ≥ 10%, IC2 ≥ 5% and < 10%, IC1 ≥ 1% and <5%, and IC0 < 1%). Patients who achieved complete remission (CR) or partial remission (PR) were classified as remission patients, and those with stable disease (SD) or disease progression (PD) were classified as non‐remitters. (B, C) Hypoxia score was different based on the differential expression of PD‐L1 on tumor and immune cells (ANOVA; ****p < 0.0001, ***p < 0.001, **p < 0.01, *p < 0.05, ns, not significant). (D)There was no significant difference observed between the hypoxia score and the state of remission and non‐remission (*t* test). (E) The hypoxia scores of the three immunophenotypes were different. Significance is denoted by asterisks (Kruskal–Wallis; ****p < 0.0001, ***p < 0.001, *p < 0.05). (F) Using Pearson's correlation analysis, the correlation between hypoxia score and other related biological processes were analyzed in the IMvigor210 cohort. Red indicates positive correlation, and blue indicates negative correlation. The larger the circle, the higher the correlation. (G) The “Submap” algorithm predicted the possibility of the hypoxia score for anti‐PD‐1 and anti‐CTLA4 immunotherapy responses (Bonferroni‐corrected, *p* < 0.01).

To further prove that the hypoxia score was effective in predicting the immunotherapy response, we used the anti‐PD‐L1 immunotherapy IMvigor210 cohort to predict the response to ICI and analyzed it using heatmap‐based joint analysis. We observed expression of PD‐L1 on immune cells, tumor cells, and response to immunotherapy with different scores. Next, we showed the distinction in TMB and mutation driver genes of BLCA in different score groups. In addition, we analyzed the differences in biological pathways between the high‐score and low‐score groups in BLCA. In immune‐related pathways such as CD8^+^ T‐effector signature, antigen processing machinery, immune checkpoint signature, the expression in the high hypoxia score group was higher than low score group. Therefore, patients with high hypoxia scores may be more suitable for immune checkpoint inhibitor therapy. The enrichment of pathways related to extracellular matrix activation such as TGF‐β receptors and ligands, Pan‐F‐TBRS genes, angiogenesis signature, and EMT markers was greater in the high‐score group than in the low‐score group. In contrast, enrichment of the terms FGFR3 gene signature, MKI67, cell cycle genes, DNA replication‐dependent histones, and DNA damage repair genes was higher in the low‐score group, Therefore, blocking these pathways may confer clinical benefit in patients with low hypoxia score (Figure 6A). In the IMvigor210 cohort, there were differences in the hypoxia scores of the tumor cells with differential expression of PD‐L1 (ANOVA, *p* = 1.4e−05; Figure 6B). This difference was also reflected by the differential expression levels of PD‐L1 in the immune cells (ANOVA, *p* = 2.7e−07; Figure 6C). However, in response to PD‐L1 blocker treatment, the difference in scores between remission (complete remission/partial remission) and non‐remission (stable disease/disease progression) groups was not significant (*t* test, *p* = 0.066; Figure 6D). Additionally, we observed differences in the hypoxia scores in different immunophenotypes; the “inflamed” phenotype had the highest score, followed by “excluded,” and finally “desert” (Kruskal–Wallis, *p* < 0.05; Figure 6E). Therefore, perhaps it is difficult for the ICIs to exert antitumor effects in non‐inflamed phenotypes, indicating that those with a high hypoxia score may show a higher association with response to anti‐PD‐L1 immunotherapy. We also evaluated the correlation between hypoxia score and BLCA biological pathways. The score has a positive correlation with CD8 T‐effector, immune checkpoint, EMT1, EMT2, and EMT3, and has a negative correlation with FGFR3, PPARG, TCGA, and DDR (Figure 6F). Finally, we used SubMap analysis to predict the immune responsiveness, and the results showed that the high‐score group may benefit more from PD‐1 treatment (Bonferroni‐corrected, *p* < 0.01; Figure 6G). In summary, the hypoxia score constructed in this study could predict the immunotherapy responsiveness of different individuals.

### 3.8 Identification and pan‐cancer analysis of the hub genes

**FIGURE 7:**
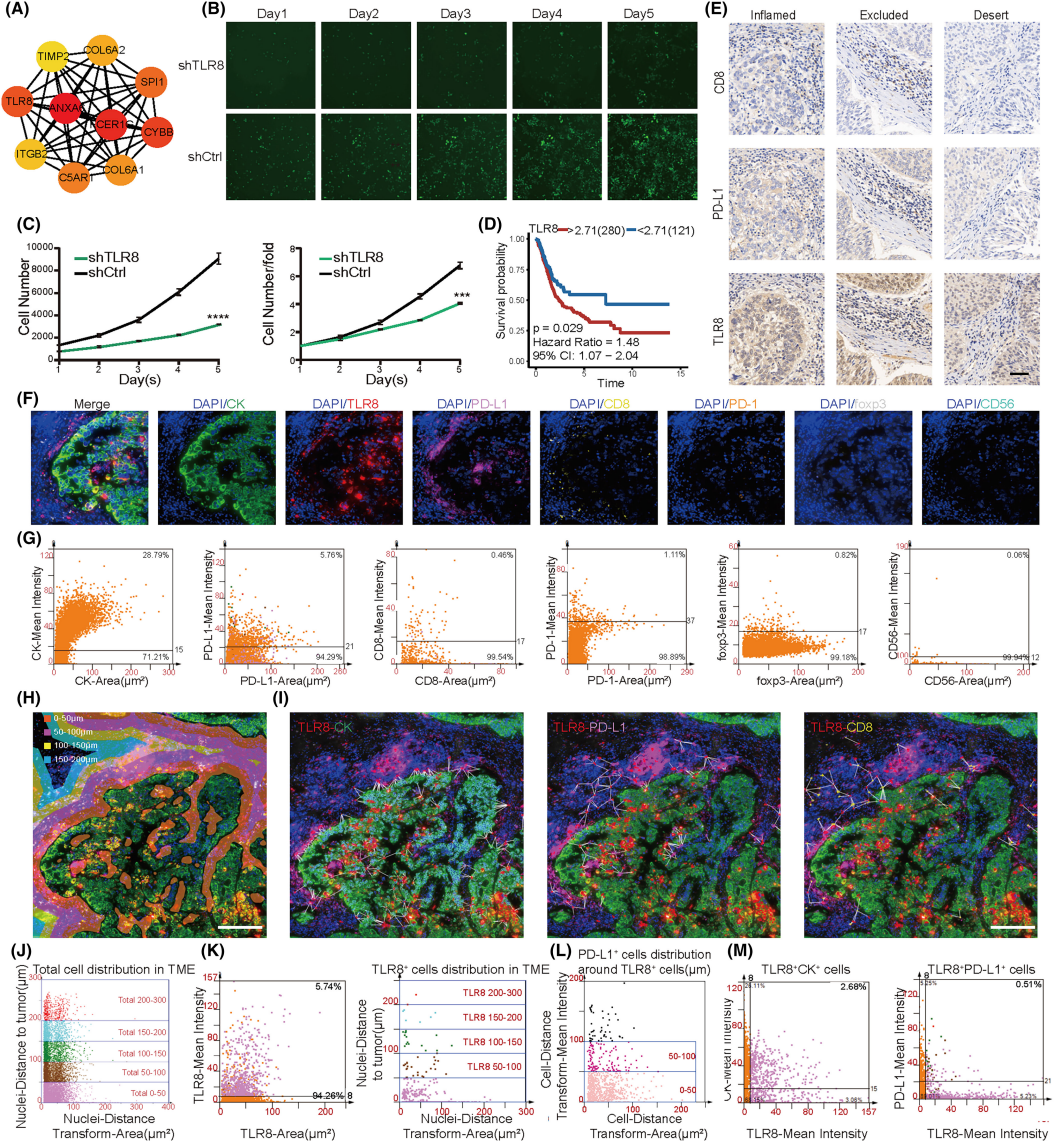
Validation of the hub genes. (A) The network of hub genes of the brown module is shown. Nodes represent the genes and node size indicate the betweenness score. (B) Fluorescence graph of *TLR8* knockdown in cell proliferation experiment at 100× magnification. (C) Line graph of cell proliferation and cell proliferation fold change after *TLR8* knockdown. (D) Kaplan–Meier curve showed that overall survival (OS) was worse when *TLR8* was highly expressed (log‐rank test, *p* = 0.029). (E) Immunohistochemistry images of the three immunophenotypes. scale bars, 50 μm. (F) Representative images for multiplexed immunofluorescence staining (DAPI, dark blue; TLR8, red; PD‐L1, purple; CD8, yellow; PD‐1, orange; foxp3, white; CD56, light blue) in BLCA. Scale bar, 100 μm. (G) The cell intensity of CK^+^ cells, TLR8^+^ cells, PD‐L1^+^ cells, CD8^+^ cells, PD‐1^+^cells, foxp3^+^ cells and CD56^+^ cells. (H) Tumor tissue was recognized by CK staining and morphology. Different colors represent different distance from tumor cells. (I) The distance of mutual neighbors was represented by white lines. scale bars, 200 μm. (J) The scatter plot shows the number of total cells at different locations from the tumor cells. (K) The intensity of TLR8^+^ cells (left image). The scatter plot shows the number of TLR8^+^ cells at different locations from the tumor cells. (L) The scatter plot shows the number of PD‐L1^+^ cells at different locations from TLR8^+^ cells. (M) The scatter plot shows the number of TLR8^+^CK^+^ cells, and TLR8^+^ PD‐L1^+^ cells.

We visualized the hub genes of the BM as a network and screened the top 10 candidate genes by ordering them as per their betweenness scores. These hub genes included *ANXA6*, *FCER1G*, *CYBB*, *TLR8*, *SPI1*, *C5AR1*, *COL6A1*, *COL6A2*, *ITGB2*, and *TIMP2* (Figure 7A). Subsequently, we analyzed these hub genes in 33 kinds of tumors using the genomic cancer analysis tool GSCALite. Using TCGA database, we compared the mRNA expression levels of the hub genes in cancerous and paired‐adjacent tissues and found a significant difference in gene expression of the hub genes in many cancers, such as lung squamous cell carcinoma and lung adenocarcinoma; in fact, most hub genes have higher expression in the normal tissues. However, in case of kidney renal cell carcinoma, most hub genes have higher expression in the cancerous tissues (Figure S6A; Table S10). Next, we determined the single‐nucleotide variant frequency of the hub genes, and observed that uterine corpus endometrial carcinoma, skin cutaneous melanoma, and colon adenocarcinoma had high mutation frequencies for most hub genes (Figure S6B). Additionally, we observed differences in the Copy number alterations (CNAs) in the pan‐cancer analysis; for instance, adrenocortical carcinoma mainly exhibited heterozygous amplification. Whereas, kidney chromophobe mainly exhibited heterozygous deletions (Figure S6C). The CNAs of the hub genes were mainly heterozygous, and homozygous changes were rare (Figure S6D,E). The above results indicated that hub genes may impact the occurrence and progression of multiple tumors. Furthermore, these analyses may accelerate the search for multi‐tumor gene markers and adjuvant immunotherapy in the future.

### 3.9 Validation of the hub genes

Patients with high expression of *ANXA6*, *CYBB*, *SPI1*, *C5AR1*, *COL6A1*, *COL6A2*, *ITGB2*, and *TIMP2* were associated with poor prognosis, indicating that these genes were possible prognostic risk factors. In contrast, *FCER1G* was a protective factor for prognosis (Figure S7A). Most hub genes activated “apoptosis,” “EMT,” “hormone ER pathways,” and inhibited “cell cycle,” “DNA damage response,” “hormone AR pathways” (Figure S7B). Next, we determined the effects of somatic cell CNAs of the hub genes on immune cell infiltration to elucidate the underlying mechanisms by which hub genes were associated with them. The CNAs of hub genes significantly affected the infiltration levels of six types of immune cells, suggesting the regulation of hub genes on TME in BLCA patients (Figure S7C). We analyzed the expression of these molecules in three different immune subtypes, and found that TLR8 is the most significant difference among the three immune subtypes(Figure S8A). Meanwhile, TLR8 has a strong correlation with CTL (Figure S8B). In the HCS, 5 days after infection, the green fluorescence signal of shTLR8‐lentivirus‐infected T24 cells was significantly weaker than that of the control cells. In addition, HCS showed that *TLR8* knockdown significantly reduced the proliferation rate of the T24 cells (fold change≧1.5, *p* < 0.05; Figure 7B,C; Figure S9A,B). Patients with high expression of *TLR8* suggested poor prognosis, indicating that *TLR8* was possible prognostic risk factors for BLCA (*p* = 0.029, HR = 1.48, 95% CI = 1.07–2.04; Figure 7D). Heatmap of the correlation between TLR8 and immune cells and immune checkpoints in multiple cancers were downloaded from the SangerBox and noted that TLR8 was closely related to immune infiltration and immunotherapy (Figure S10A,B). Samples were classified to three immune phenotypes, “inflamed,” “excluded,” and “deserted.” We observed that CD8, PD‐L1, and TLR8 were more distributed in the “inflamed” phenotype and “excluded” phenotype. Representative images of immunohistochemistry were shown in Figure 7E and the corresponding statistical chart is shown in Figure S11. To explore the potential interactions between TLR8 and immune cell infiltration a step further, multispectral immunofluorescence staining was to estimate the expression pattern of CK, TLR8, PD‐L1, CD8, PD‐1, foxp3, and CD56. The spatial distribution of TLR8 and PD‐L1 represented a similar pattern. Compared to CD8^+^ cells accumulated in stromal region, TLR8^+^ cells and PD‐L1^+^ cells were more accumulated in parenchymal/tumor region (Figure 7F). At the same time, we calculated the cell intensity of CK^+^ cells (28.79%), PD‐L1^+^ cells (5.76%), CD8^+^ cells (0.46%), PD‐1^+^ cells (1.11%), foxp3^+^ cells (0.82%), and CD56^+^ cells (0.06%) (Figure 7G). Calculating the number of tumor cells at different distance gradients, we found that the further away from the tumor nest, the overall cells gradually decreased (Figure 7H,J). The same result can also be found in TLR8^+^ cells (cell intensity 5.74%) (Figure 7K). We used the topographic information derived from multispectral imaging and estimated the mutual neighbor distances between each cell pair (TLR8^+^ and CK^+^, TLR8^+^ and PD‐L1^+^, TLR8^+^, and CD8^+^). Consistent with the distribution pattern, the mutual neighbor distance between TLR8^+^ cells and CK^+^ cells is shortest. The distance of TLR8^+^ cell and PD‐L1^+^ cell was shorter than TLR8^+^ cell and CD8^+^ cell (Figure 7I). In addition, Number of PD‐L1^+^ cells gradually decreased as the distance from TLR8^+^ cells got farther (Figure 7L). TLR8^+^CK^+^ cells (2.68%) and TLR8^+^PD‐L1^+^ cells (0.51%) were also found in bladder cancer tissues (Figure 7M). Collectively, TLR8 is associated closely with biomarkers of immune infiltration, especially PD‐L1. In summary, TLR8 was closely associated with the cellular malignant phenotype, immune infiltration, and immunophenotyping of BLCA, and is thus, a potential target for immunotherapy.

## 4 DISCUSSION

Advances in immunotherapy have revolutionized the treatment strategies for BLCA. Hypoxia affects the TME and efficacy of immunotherapy. Gene signatures related to hypoxia can be used to determine the response of individuals to ICIs and provide guidance for performing immunotherapy in patients with BLCA.

Herein, we classified the patients with BLCA into two clusters based on expression of hypoxia‐related genes in brown module. We found that the molecular subtype in this study can be a good complement to the current classical classification of bladder cancer. For example, bladder cancer patients with basal‐like features have worse tumor progression outcomes and worse prognosis. Genes involved in the biological processes of immune and inflammatory responses were also significantly enriched in basal‐like bladder cancer, revealing that basal subtype may be a candidate for immune checkpoint therapy.^30^ This is consistent with the prognostic and immune‐related analysis of the subtype in this paper. We comprehensively calculate the infiltration levels of the immune cells and observed that immune infiltration in Cluster1 was significantly stronger than that of Cluster2. Patients with high immune‐infiltrated tumors demonstrated higher response rates to treatment and improved survival rates. Enhancing immune infiltration generates systemic CD8^+^ T cell‐mediated antitumor immunity and sensitizes resistant tumors to checkpoint blockade.^53^ Cluster1 was more closely related to the CD8 T‐effector signature and immune checkpoints. As previously mentioned, the response to treatment was associated with the CD8^+^ T‐effector cell phenotype, and the use of blockers against immune checkpoints provides meaningful clinical benefits.^11^ The signaling pathways of DEGs of two clusters play key roles during immune response, immune surveillance, and immune escape. IL‐6/JAK/STAT3 signaling can drive tumor cell proliferation, invasion, and metastasis in the TME, and strongly inhibit the antitumor immune response.^54^ CNAs are an independent predictor of ICI treatment in cancer predictive analysis.^55^ The differences in the genomic variations between Cluster1 and Cluster2 also confirmed the differences in immune infiltration. Therefore, our results revealed BLCA subtypes had a considerable effect on predicting the immunotherapeutic response.

Considering the individual heterogeneity of individual patients with malignant tumors, it is urgent to quantify the TME‐related immune cell infiltration characteristics in individual tumors. Therefore, here, we developed a risk scoring system (hypoxia score) based on hypoxia‐related genes and performed external validation for its performance using IMvigor210 cohort. Patients in the high hypoxia score group presented higher levels of immune infiltration. In the groups, we observed a significant difference between the PD‐L1 expression level on immune cells and PD‐L1 expression level on tumor cells. The infiltration characteristics of the TME cells were consistent with the three tumor immunophenotypes and can be used to predict tumor inflammation stages, subtypes, TME stromal activity, genetic variation, patient prognosis, and immunotherapy responsiveness.^56^ Several studies have suggested that increased PD‐L1 expression on tumor cells and immune cells may be an independent predictor of enhanced clinical benefits of atezolizumab use.^57^, ^58^ In addition, Patients with inflammatory tumors with high PD‐L1 levels have higher 5‐year survival rates.^59^ The hypoxia score was able to distinguish the three immunophenotypes, wherein, the “inflamed” phenotype had the highest score and the “desert” phenotype had the lowest score, which is same to the results of previous studies. To enhance the reliability of immune response prediction, we used the SubMap algorithm and observed that patients with high hypoxia score had better immunotherapy response, confirming the role of the hypoxia score.

Interestingly, there were no significant differences in the immunotherapy remission rates in patients with different scores. Cluster1 and high‐score groups had stronger immune infiltration; however, survival analysis showed worse prognosis, which may be owing to the tumor immune escape mechanism, which is related to tumor immune dysfunction and exclusion.^60^ There is T‐cell dysfunction in tumors with high infiltration of cytotoxic T lymphocytes, and prevention of T‐cell infiltration in tumors with low CTLs. Some tumors show a high level of infiltration by cytotoxic T cell. However, these T cells tend to be in a dysfunctional state. In other tumors, immunosuppressive factors may exclude the T cells from infiltrating the tumors.^61^ Pérez‐Guijarro et al. have demonstrated that there is an apparent correlation between T‐cell dysfunction, exclusion procedures, and tumor drug resistance; the combination of these characteristics can better predict the response to ICI. The results of their study also underscored the importance of T‐cell functionality, and the complexity of ICI responses as the combination of immune‐related and tumor cell‐intrinsic factors demonstrated improved performance in patient outcome prediction.^62^

We used Cytoscape and screened 10 hub genes from the BM. Most of the identified hypoxic hub genes have been associated with immune processes. For example, the combination of immunogenic cell death and activation of TLR8 enhances the maturation of dendritic cells, which amplifies antitumor immune responses.^63^ TLR8 is highly expressed on Tregs, and its ligands can reverse the immunosuppressive function of Tregs.^64^ TLR8 played crucial role in initiating innate and adaptive immune responses. TLR8 pathways is favorable targets for immunological therapies.^65^ B cell‐extrinsic *FCER1G* negatively regulates autoreactive and normal B‐cell immune responses.^66^ CYBB‐regulated antigen processing and LC3‐associated phagocytosis in conventional dendritic cells enables encephalitogenic Th cells to initiate and sustain autoimmune neuroinflammation.^67^ Thus, these hypoxia hub genes are expected to be valuable targets for immunotherapy. TLRs are widely expressed in different types of tumor cells and play different roles.^68^ Some Toll‐like receptor ligands, such as imiquimod, a TLR7 agonist, and CpG, a TLR9 ligand, can directly induce apoptosis of TLR positive tumor cells, or enhance tumor‐infiltrating innate immune cells and tumor specific T‐cell functions.^69^, ^70^, ^71^, ^72^, ^73^, ^74^ On the contrary, TLR2 and TLR5 signal pathways can enhance the proliferation and survival of gastric cancer cells and promote tumor migration.^75^, ^76^ Recent studies have shown that human tumor cells can transform initial/effector T cells into senescent T cells to induce immune tolerance, which depends to some extent on the endogenous metabolic cAMP of tumor origin. It is worth noting that activating TLR8 signal in tumor cells can prevent tumor cells from producing CAMP, reverse the transformation of tumor‐induced initial type, and tumor‐specific T cells into senescent cells, and thus enhance antitumor immunity in vivo.^77^, ^78^ At the same time, studies have shown that TLR1/2 and TLR8 can reverse the inhibition of Treg by regulating Treg metabolism.^76^, ^77^, ^78^, ^79^, ^80^, ^81^ These previous studies have emphasized the important role of TLR8 in tumor immune microenvironment. Previous studies on TLR8 mainly focused on breast cancer, melanoma, prostate cancer, and other models, and rarely involved bladder cancer. Gianmarco Garau et al. Found that the expression of TLR8 in muscle invasive bladder cancer (MIBC) was higher than that in non‐muscle invasive bladder cancer (NMIBC) by immunohistochemistry on the pathological sections of 25 patients undergoing transurethral cystectomy (turb).^82^ The expression of TLR8 in high‐grade (Hg) bladder cancer was higher than that in low‐grade (LG) bladder cancer. These early studies around clinical samples of bladder cancer verified the potential poor prognosis of TLR8 in bladder cancer. In our findings, the cell proliferation assay showed that *TLR8* knockdown inhibited the proliferation of the BLCA cells. Immunohistochemistry showed that CD8, PD‐1, and TLR8 were more expressed in the “inflamed” and “excluded” phenotype. Multispectral immunofluorescence suggested that TLR8 is associated closely with biomarkers of immune infiltration, especially PD‐L1. Overall, TLR8 should be investigated as a predictive biomarker for tumor immune infiltration and immunotherapy; however, the mechanism of action of TLR8 in immunotherapy needs to be further explored.

In this study, we have provided novel insight into the possibility of immunotherapy as a therapeutic modality for BLCA, and suggested hypoxia‐related genes that may contribute to the development of new strategies or new immunotherapeutic agents in the future. Our newly constructed subtypes and hypoxia score suggest potential treatment guidelines for patients with BLCA, especially in terms of ICI response. TLR8 can also be used as a potential target for immunotherapy.

We have provided some novel insights for bladder cancer immunotherapy based on hypoxia‐related genes, which may help to develop novel drug combination strategies or immunotherapeutic agents in the future. Our newly constructed molecular subtype and hypoxia score suggest potential treatment guidelines for each patient with bladder cancer, especially with regard to responsiveness to immune checkpoint inhibitors. However, the limitations of the study must be acknowledged. Our study must be prospectively validated before being put into clinical implementation. More clinical patient data and pathological specimens need to be collected for validation. All the experiments in this study were carried out in vitro, and thus, in vivo studies and more experiments based on cell lines are required to confirm our findings. Research techniques such as single‐cell sequencing and spatial transcriptomics also allow for unprecedented levels of heterogeneity in bladder cancer, which will be the focus of our future research.

## 5 CONCLUSIONS

In conclusion, this study proved that hypoxia affect immune infiltration in the TME, and hypoxia‐based markers can guide more effective immunotherapy strategies. The BLCA subtypes and hypoxia score, based on hypoxia‐related genes, could effectively predict therapeutic responses in patients with BLCA. Furthermore, the hypoxia‐related gene TLR8 can be a potential target for immunotherapy. Taken together, our results make a significant contribution to literature as they can guide more effective immunotherapeutic strategies in BLCA.

## AUTHOR CONTRIBUTIONS

**Haotian Chen:** Data curation (equal); formal analysis (equal); methodology (equal); software (equal); writing – original draft (equal). **Yao Zhang:** Data curation (equal); formal analysis (equal); investigation (equal); methodology (equal); software (equal); writing – original draft (equal). **Xingyu Chen:** Data curation (supporting); formal analysis (supporting); software (supporting); validation (supporting); visualization (supporting); writing – review and editing (supporting). **Runshi Xu:** Formal analysis (supporting); writing – review and editing (supporting). **Yuxing Zhu:** Writing – review and editing (supporting). **Dong He:** Writing – review and editing (supporting). **YaXin Cheng:** Writing – review and editing (supporting). **Zhanwang Wang:** Writing – review and editing (supporting). **Ke Cao:** Funding acquisition (supporting); project administration (supporting); resources (supporting).

## FUNDING INFORMATION

This work was supported by the National Natural Science Foundation of China (81874137), Funds for International Cooperation and Exchange of the National Natural Science Foundation of China (GZ1699), key research and development projects in Hunan Province (2022SK2022), the science and technology innovation Program of Hunan Province (2020RC4011), the Hunan Province Science and Technology Talent Promotion Project (2019TJ‐Q10), Scientific research project of Hunan Provincial Health Commission (202209034683), Young Scholars of “Furong Scholar Program” in Hunan Province, and the Wisdom Accumulation and Talent Cultivation Project of the Third xiangya hospital of Central South University (BJ202001).

## CONFLICT OF INTEREST STATEMENT

The authors declare no competing interests.

## Supporting information


Figure S1.

Figure S2.

Figure S3.

Figure S4.

Figure S5.

Figure S6.

Figure S7.

Figure S8.

Figure S9.

Figure S10.

Figure S11.

Table S1.
Table S2.
Table S3.
Table S4.
Table S5.
Table S6.
Table S7.
Table S8.
Table S9.
Table S10.
Table S11.

## Data Availability

Not applicable.
