# Supplementary figures and images for: Hypoxia is correlated with the tumor immune microenvironment: Potential application of immunotherapy in bladder cancer
Source: Cancer Med. 2023 Dec 8;12(24):22333–53. doi: 10.1002/cam4.6617 (PMC10757107; doi:10.1002/cam4.6617)

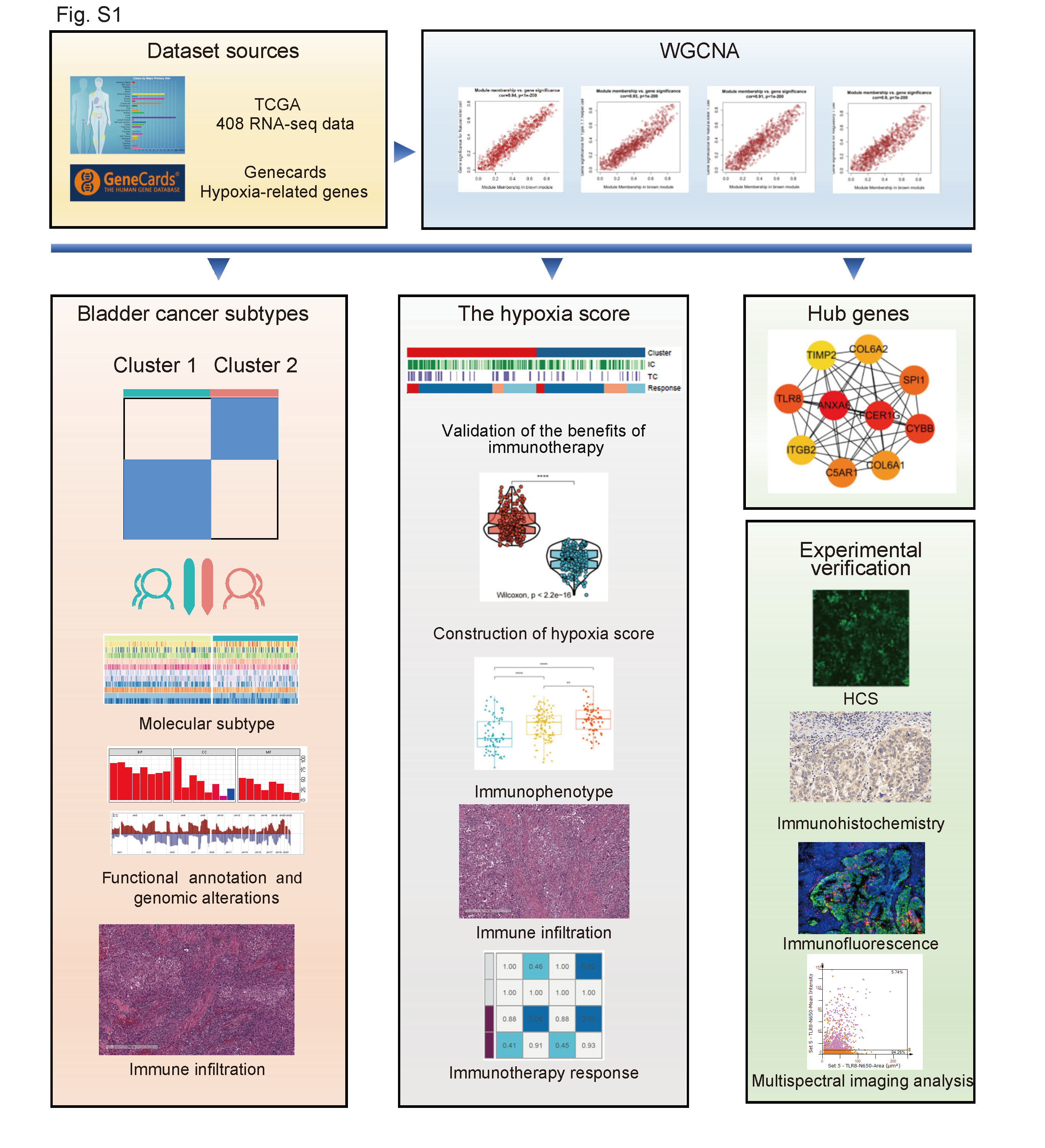

Supplement: Supplementary file 1 — Figure S1. [file CAM4-12-22333-s008.tif]

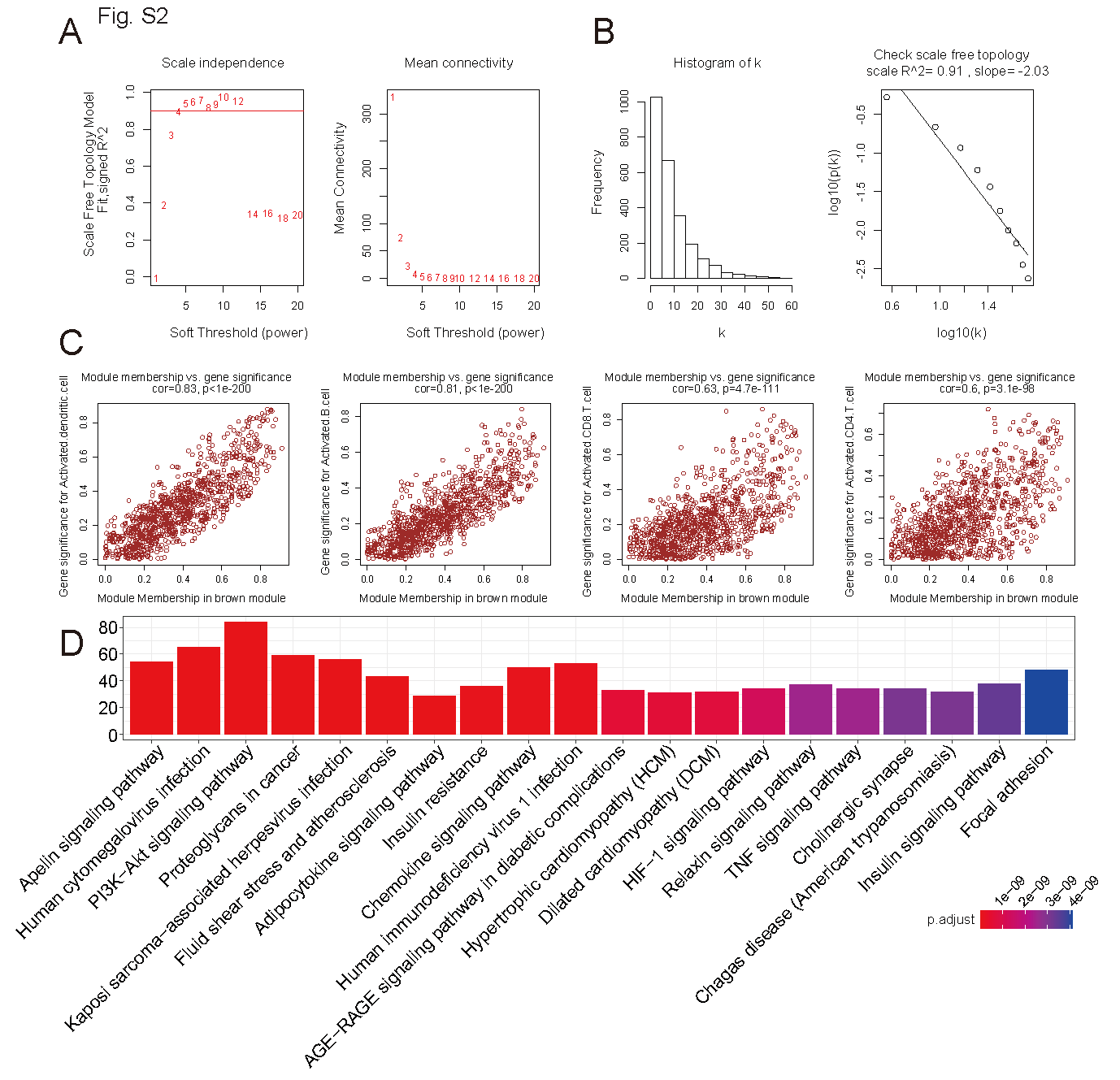

Supplement: Supplementary file 2 — Figure S2. [file CAM4-12-22333-s011.tif]

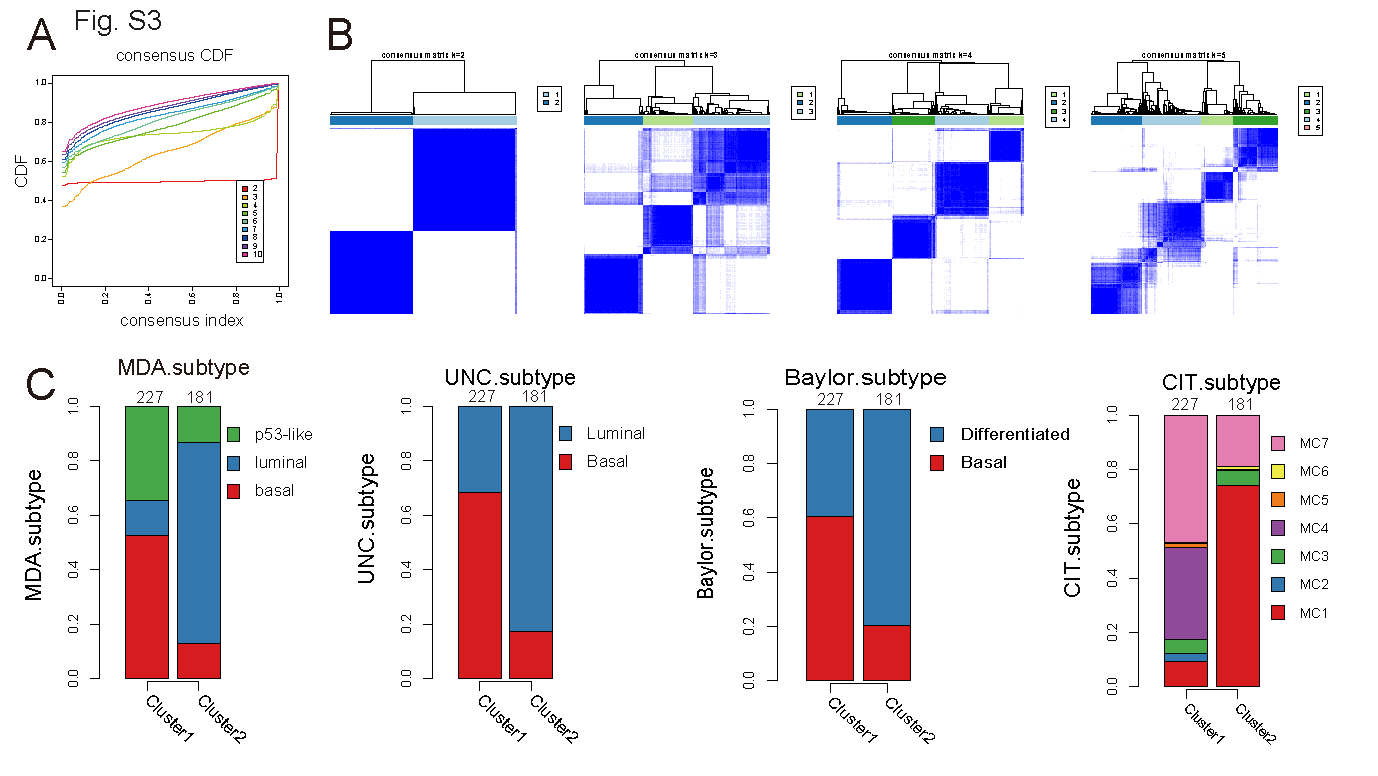

Supplement: Supplementary file 3 — Figure S3. [file CAM4-12-22333-s002.tif]

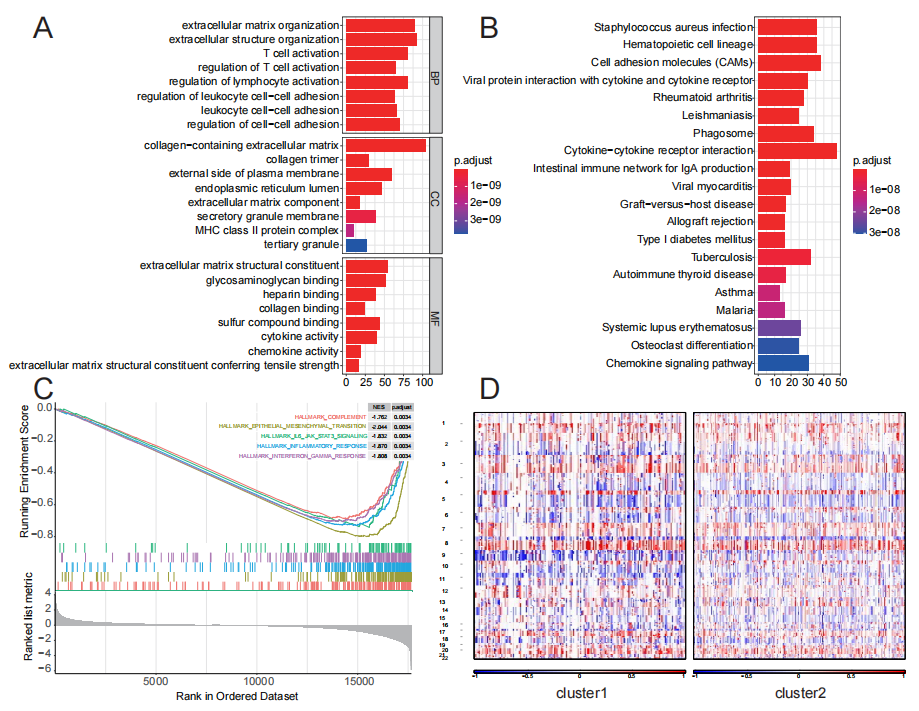

Supplement: Supplementary file 4 — Figure S4. [file CAM4-12-22333-s005.tif]

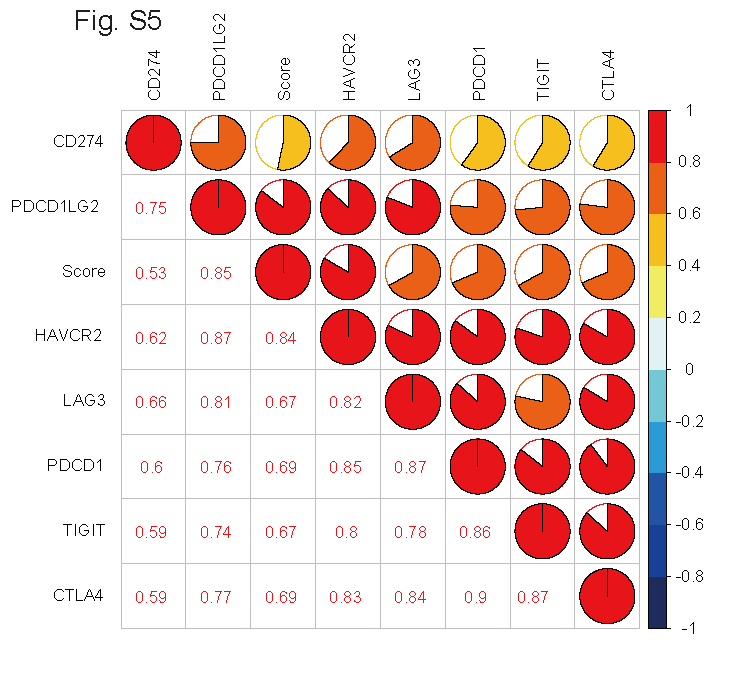

Supplement: Supplementary file 5 — Figure S5. [file CAM4-12-22333-s001.tif]

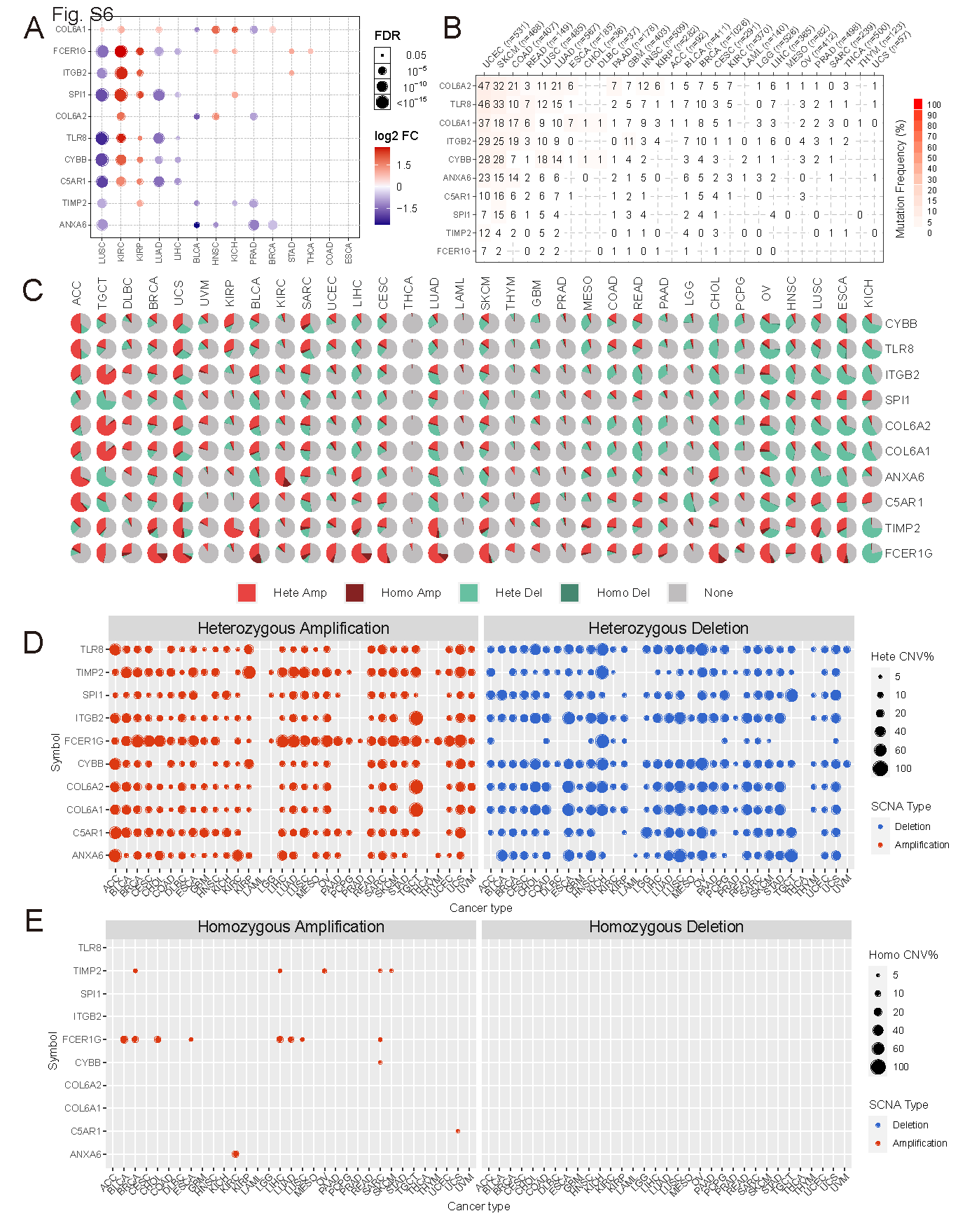

Supplement: Supplementary file 6 — Figure S6. [file CAM4-12-22333-s009.tif]

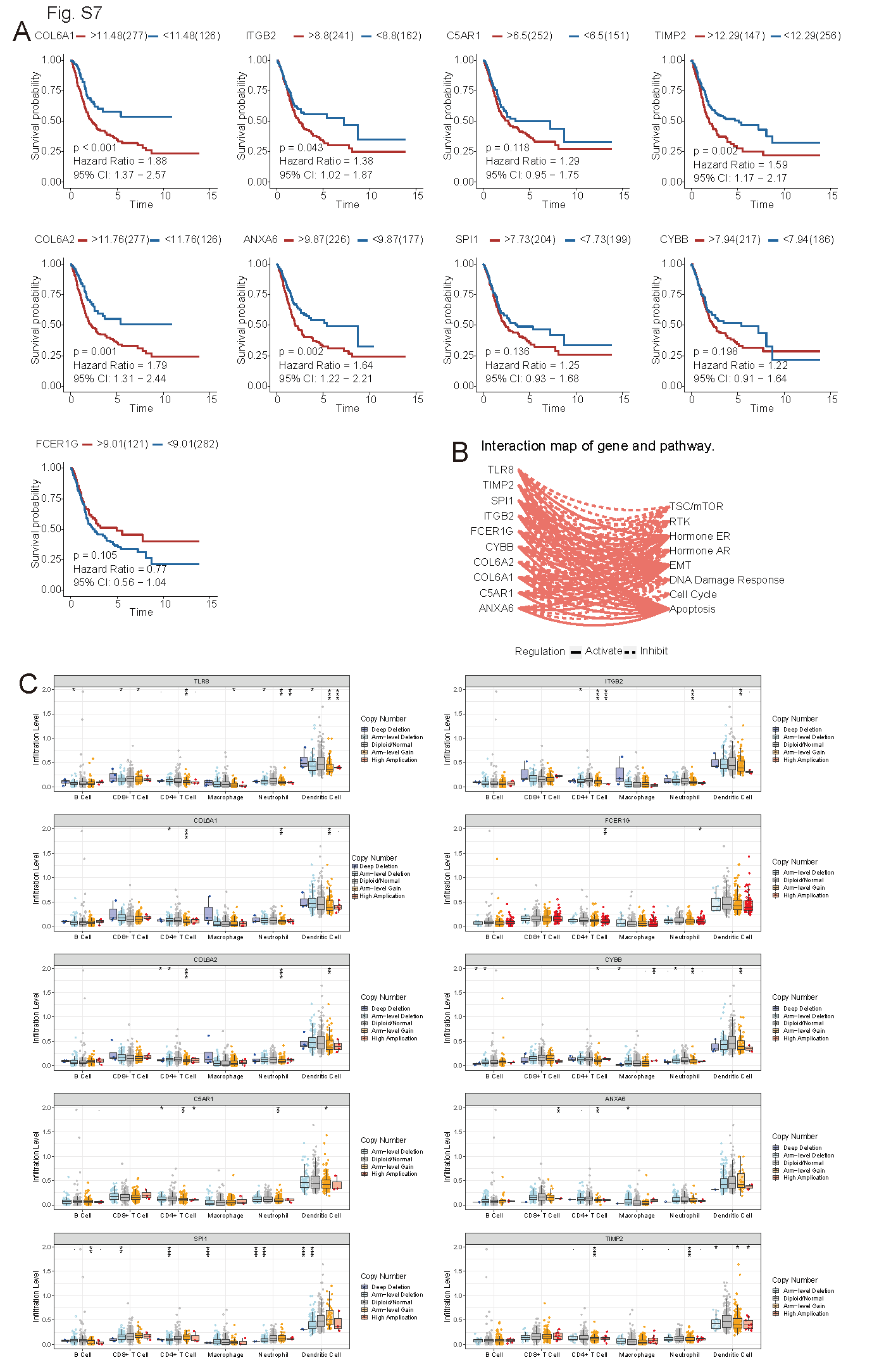

Supplement: Supplementary file 7 — Figure S7. [file CAM4-12-22333-s006.tif]

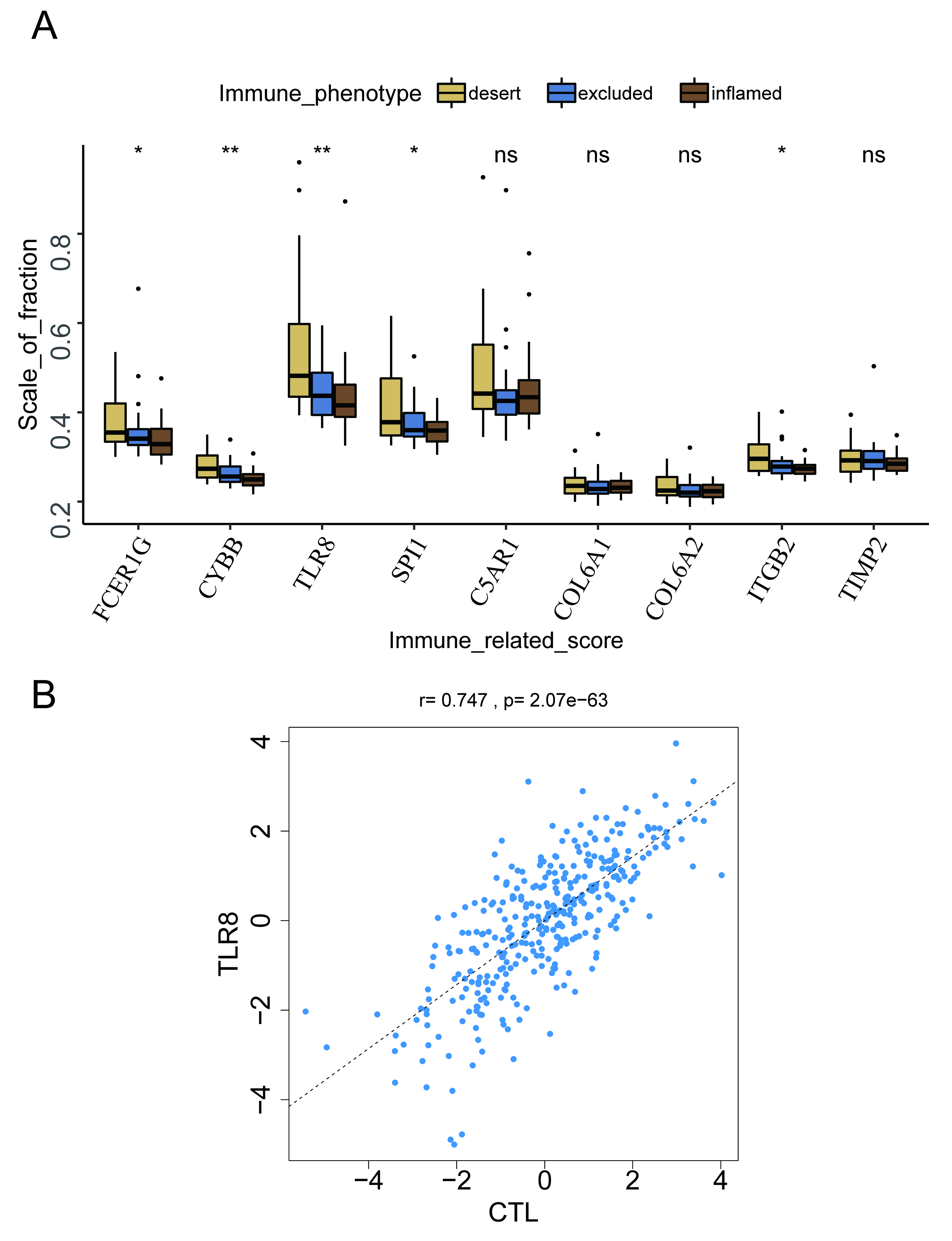

Supplement: Supplementary file 8 — Figure S8. [file CAM4-12-22333-s003.tif]

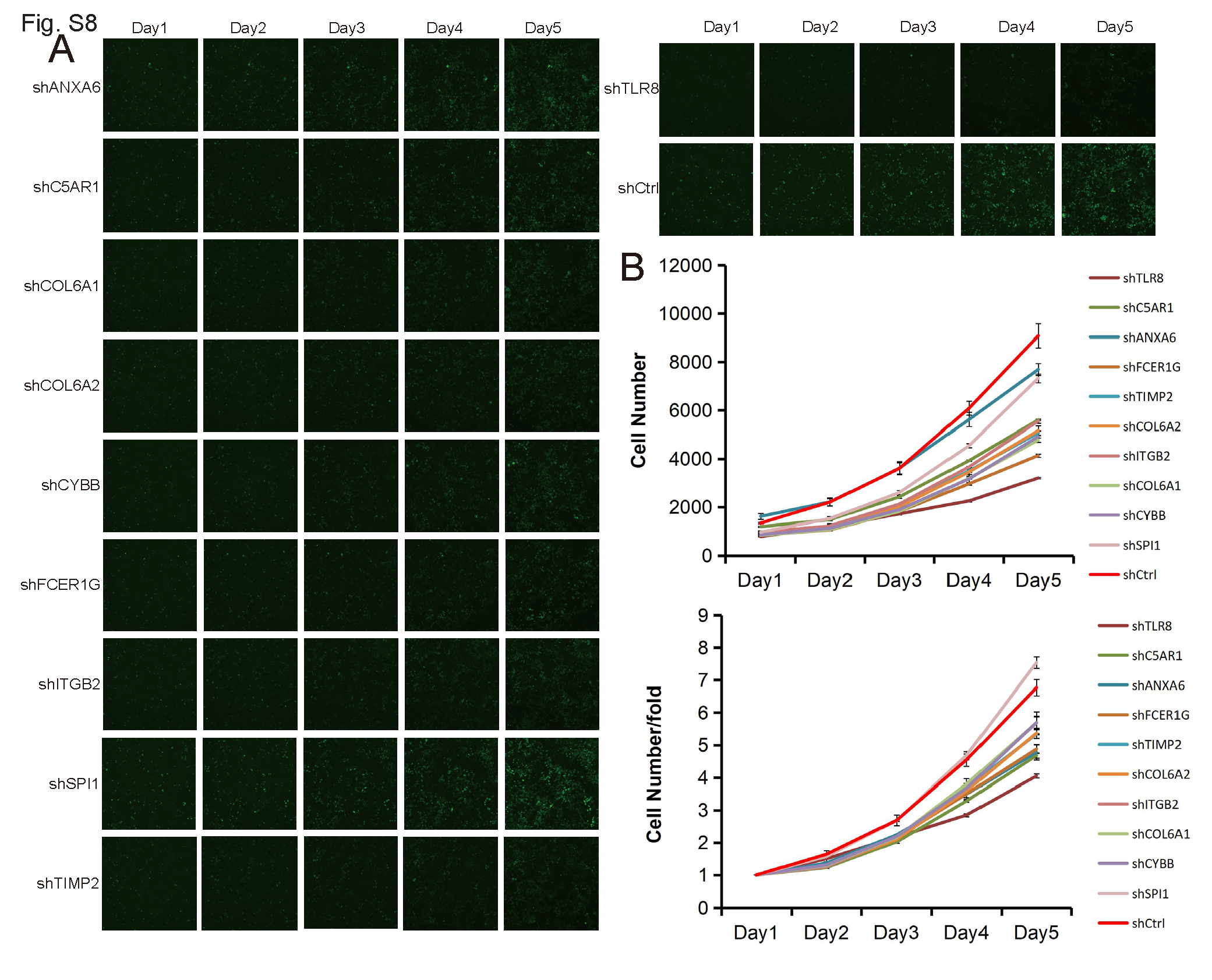

Supplement: Supplementary file 9 — Figure S9. [file CAM4-12-22333-s007.tif]

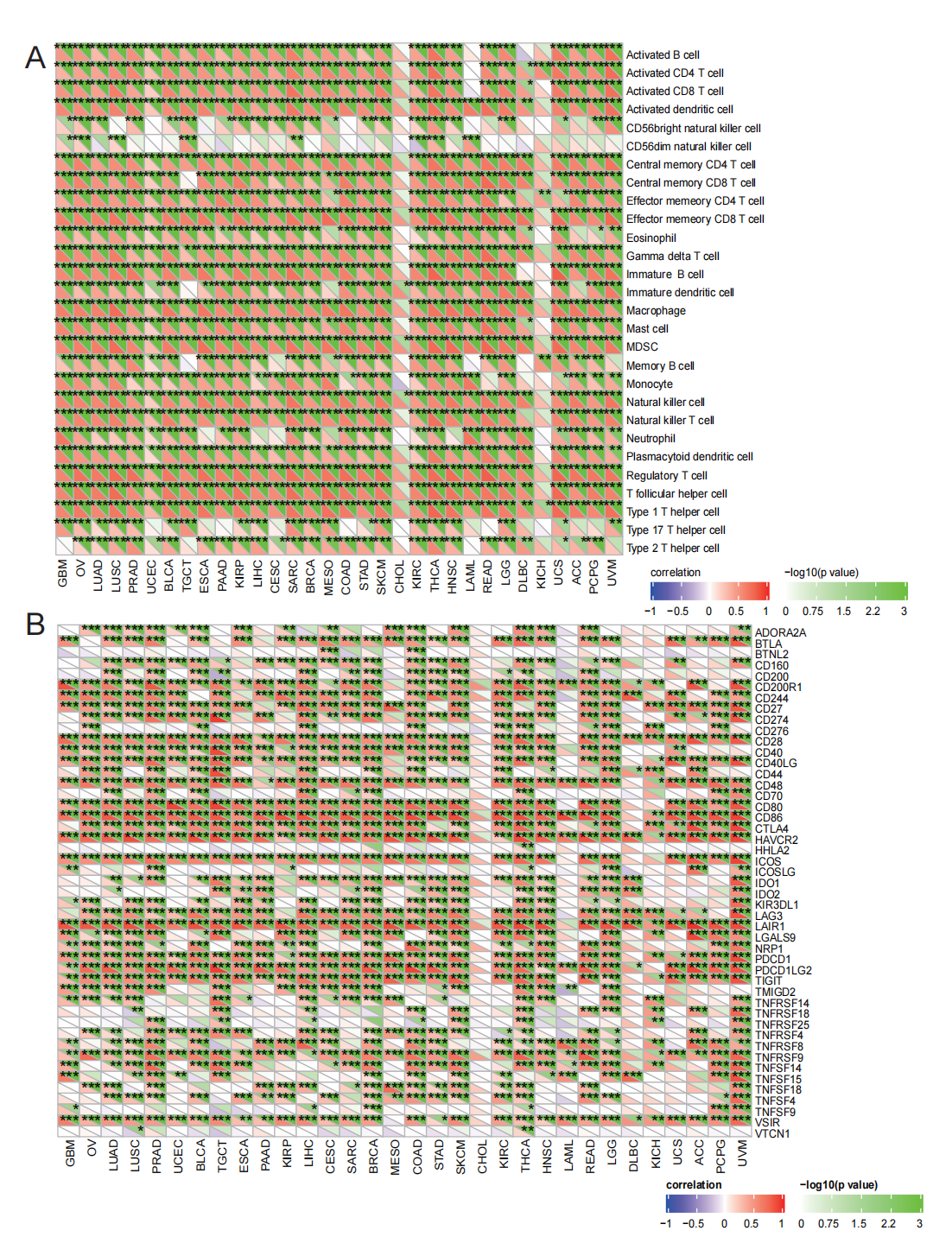

Supplement: Supplementary file 10 — Figure S10. [file CAM4-12-22333-s004.tif]

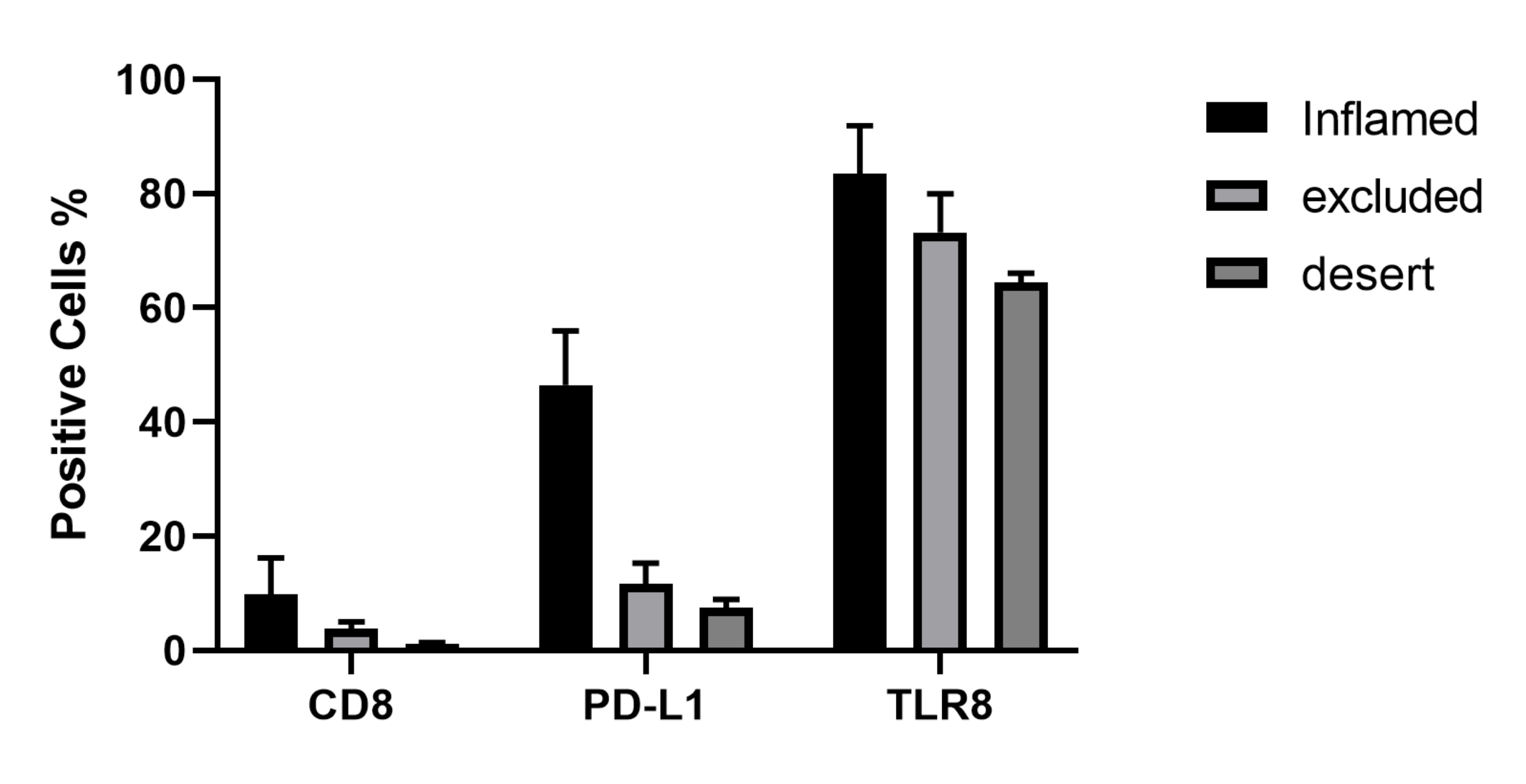

Supplement: Supplementary file 11 — Figure S11. [file CAM4-12-22333-s010.tif]
