# Supplementary material for: Hypoxia is correlated with the tumor immune microenvironment: Potential application of immunotherapy in bladder cancer
Source: Cancer Med. 2023 Dec 8;12(24):22333–53. doi: 10.1002/cam4.6617 (PMC10757107; doi:10.1002/cam4.6617)
Supplement: Supplementary file 12 — Table S1. Table S2. Table S3. Table S4. Table S5. Table S6. Table S7. Table S8. Table S9. Table S10. Table S11. [file CAM4-12-22333-s012.zip › Supplementary captions.docx]

Thank you very much! Below are the names of Supplementary Tables caption.

①the caption of Supplementary Table S1 is "Detailed clinical information of patients from TCGA-BLCA database"

②the caption of Supplementary Table S2 is "Details of the samples from the IMvigor210 cohort"

③the caption of Supplementary Table S3 is "The sequence information of shRNA used in this experiment"

④the caption of Supplementary Table S4 is "GO analysis of the brown module"

⑤the caption of Supplementary Table S5 is "KEGG analysis of brown module"

⑥the caption of Supplementary Table S6 is "GO analysis of BLCA subtypes"

⑦the caption of Supplementary Table S7 is "KEGG analysis of BLCA subtypes"

⑧the caption of Supplementary Table S8 is "GSEA analysis of BLCA subtypes"

⑨the caption of Supplementary Table S9 is "Clinical stage of BLCA subtypes and hypoxia score"

⑩the caption of Supplementary Table S10 is "Differential expression of key genes

between tumor and normal"

**⑪**the caption of Supplementary Table S11 is "The  Gene ID of Brown Module"
